# Xinnaoxin tablets ameliorate high-altitude polycythemia-associated cardiac injury by regulating the NF-κB, MAPK, and PI3K/AKT signaling pathways

**DOI:** 10.3389/fphar.2026.1754806

**Published:** 2026-05-28

**Authors:** Zhihui Wang, Bing Cui, Yaqi Zhang, Lina Liu, Xinzhong Li, Min Wang, Guibo Sun

**Affiliations:** 1 Institute of Medicinal Plant Development, Chinese Academy of Medical Sciences & Peking Union Medical College, Beijing, China; 2 Jing Zhongshan Sinopharm (Tangshan) Co., Ltd., Tangshan, Hebei, China; 3 School of Pharmacy, Harbin University of Commerce, Harbin, China

**Keywords:** Xinnaoxin, hypobaric hypoxia, high-altitude polycythemia, mitogen-activated protein kinase, inflammation, apoptosis

## Abstract

**Background and Objective:**

High-altitude polycythemia (HAPC) and its associated cardiac complications, induced by hypobaric hypoxia (HH), pose significant clinical challenges. Xinnaoxin (XNX) tablets are clinically utilized for these conditions; however, their integrated multi-target mechanisms remain poorly understood. This study aims to elucidate the novel mechanisms and therapeutic potential of XNX against HAPC and HH-induced cardiac injury. For the first time, we employed a combined strategy of systems pharmacology and multi-level analysis to comprehensively investigate how XNX confers synergistic protection by modulating both the hematopoietic microenvironment and myocardial signaling networks.

**Methods:**

The metabolites of XNX were systematically identified, and its chemical profile was established using UPLC-Q-TOF-MS. An HH mouse model was generated by simulating a high-altitude hypoxic environment. Comprehensive assessments included complete blood parameters, hemorheology, the proportion and apoptosis of CD71^+^ bone marrow cells, and serum levels of erythropoietin (EPO) and pro-inflammatory cytokines (TNF-α, IL-1β, IL-6). Cardiac injury was evaluated through histopathology, echocardiography, oxidative stress indicators (MDA, T-AOC, CAT, SOD), and Western blot analyses of key signaling pathways, including phosphorylation status of MAPK, JNK, ERK, IκBα, NF-κB, and Akt, as well as the expression of apoptosis-related proteins Bax and Bcl-2.

**Results:**

XNX significantly reversed HH-induced elevations in red blood cell count, hemoglobin, hematocrit, white blood cell count, and plasma viscosity, while reducing serum EPO levels. Notably, XNX decreased the bone marrow population of CD71^+^ cells, indicating inhibition of “ineffective erythropoiesis.” Regarding cardioprotection, XNX markedly alleviated myocardial injury, reduced oxidative stress (MDA), enhanced antioxidant enzyme activities, and suppressed pro-inflammatory cytokine release. Mechanistically, XNX coordinately modulated the phosphorylation levels of multiple signaling pathways (MAPK, JNK, IκBα, NF-κB, Akt) and regulated the Bax/Bcl-2 balance, thereby creating a signaling environment favorable for cardiomyocyte survival and repair.

**Conclusion:**

XNX exerts its therapeutic effects through a dual mechanism: (1) ameliorating HAPC at its source by regulating EPO expression and enhancing bone marrow erythropoietic efficiency, and (2) counteracting HH-induced cardiac injury via multi-target modulation of the MAPK-related signaling network. These findings clarify the pharmacological basis of XNX and provide a theoretical foundation for developing multi-pathway synergistic therapies for high-altitude hypoxia-related cardiovascular diseases.

## 1 Introduction

The hypobaric-hypoxic (HH) environment characteristic of high-altitude regions can lead to chronic mountain sickness (CMS). Epidemiological studies report that the global incidence of CMS ranges from 5% to 33% (Zhao et al., 2012). CMS is primarily characterized by significant polycythemia. Research indicates that individuals residing at high altitudes who develop high-altitude polycythemia (HAPC) are at a substantially increased risk of cardiovascular complications compared to healthy individuals (Corante et al., 2018). Prolonged exposure to high-altitude conditions reduces arterial oxygen content, prompting the body to increase red blood cell (RBC) production as a compensatory response. This elevation in RBC concentration raises blood viscosity, predisposing individuals to pulmonary hypertension, heart failure, and related cardiovascular disorders. Importantly, both inflammation and cardiomyocyte apoptosis play central roles in the progression of right ventricular (RV) hypertrophy and subsequent heart failure (Ryan et al., 2015; Crawford et al., 2017; Sharifi Kia et al., 2020). Therefore, therapeutic strategies that target anti-inflammatory and anti-apoptotic pathways are considered essential for preventing the advancement of right heart failure (Bogaard et al., 2009; Sun et al., 2018; Gao et al., 2022).

Xinnaoxin (XNX), developed by Professor Zhang Xinsheng, is a formulation composed of three botanical drug s: the Tibetan botanical drug Rhodiolae Crenulatae Radix et Rhizoma [Rhodiola crenulata (Hook. f. & Thomson) H. Ohba, (Crassulaceae)], Hong Jing Tian, sourced from Qinghai Province; the Chinese botanical drug Lycii Fructus [*Lycium barbarum* L., (Solanaceae)]; and the fresh pulp of Hippophae Fructus [*Hippophae rhamnoides* L., (Elaeagnaceae)] (www.worldfloraonline.org). Within the framework of traditional Chinese medicine (TCM), erythrocytosis and heart failure are primarily attributed to the syndrome of deficiency of both Qi and Yang, accompanied by blood stasis. Accordingly, the therapeutic strategy of XNX is based on the principles of warming yang, invigorating Qi, and activating blood circulation, consistent with its traditional use. XNX is a licensed proprietary Chinese herbal medicine approved by China’s national medical products administration and is the only cardiovascular drug listed in China’s national reimbursement drug list that simultaneously supplements both Yin and Yang. According to the *Pharmacopoeia of the People’s Republic of China*, XNX is traditionally used to treat palpitations, asthma, and fatigue caused by qi deficiency and blood stasis, as well as polycythemia induced by hypoxia. Clinically, it is employed to manage HAPC, chronic pulmonary heart disease, ischemic cerebrovascular disease, and other cardiovascular disorders. Recent studies have begun to elucidate the modern pharmacological effects of XNX. Extracts of the traditional Tibetan medicine *Rhodiola rosea* have been shown to alleviate altitude sickness by increasing blood oxygen levels, scavenging free radicals, preventing hemorheological abnormalities, and improving cardiac function (Hou et al., 2024; Gao et al., 2025). Similarly, Hippophae Fructus [*Hippophae rhamnoides* L., (Elaeagnaceae)] has been reported to ameliorate hemodynamic and hematological disturbances and improve RV morphology in HAPC model rats (Zhou et al., 2012). XNX has also demonstrated neuroprotective effects and mitigated pathology in Alzheimer’s disease models, potentially via antioxidant and anti-inflammatory mechanisms (Wu et al., 2025). Notably, clinical studies have indicated that XNX can increase plasma erythropoietin (EPO) levels and improve outcomes in heart failure (Gou et al., 2016).

We established a mouse model of HH-induced polycythemia accompanied by RV injury to evaluate the therapeutic potential of XNX. Administration of XNX effectively ameliorated HH-induced polycythemia and improved RV function. However, the precise molecular mechanisms underlying these effects remain unclear. This study aimed to systematically investigate the therapeutic effects of XNX on HH-induced HAPC and associated cardiac injury, as well as the potential molecular pathways involved. The HH model was validated through comprehensive assessment of complete blood count (CBC) and hemorheological parameters. Flow cytometric analyses of bone marrow and peripheral blood revealed altered cellular distributions, including reduced apoptosis of reticulocytes and modulated erythroid maturation. The polycythemic response observed in HAPC was associated with increased erythropoietic activity in the bone marrow coupled with suppression of erythrocyte apoptosis. To explore possible underlying mechanisms, we employed network analysis as a hypothesis-generating tool to identify pathways of interest. These predicted pathways were subsequently validated using ELISA and Western blot analyses, which suggested that XNX may mitigate HH-induced cardiac injury by inhibiting oxidative stress, inflammation, and apoptosis in cardiomyocytes.

## 2 Methods

### 2.1 Reagents

The metabolites and contents of XNX tablets are presented in Table 1. XNX tablets were purchased from Jingzhongshan (Tangshan) and included in the National Medical Products Administration of China (approval number: Z20090113, batch number: 240202). Acetazolamide (ACZ) was purchased from Yunnan Pharmaceutical Co., Ltd. MAPK rabbit pAb (Cat. A5049, ABclonal Biotechnology Co., Ltd.), p38 MAPK rabbit mAb (Cat. AP1311, ABclonal Biotechnology Co., Ltd.), JNK1/2/3 rabbit mAb (Cat. A4867, ABclonal Biotechnology Co., Ltd.), phospho-JNK1/2/3 rabbit mAb (Cat. APP0631, ABclonal Biotechnology Co., Ltd.), AKT rabbit antibody (Cat. 10176-2, Proteintech Group, Inc.), phospho-AKT (Ser473) monoclonal antibody (Cat. 66444-1, Proteintech Group, Inc.), ERK1/2 rabbit mAb (Cat. 4782, ABclonal Biotechnology Co., Ltd.), phospho-ERK1/2 rabbit mAb (Cat. AP0974, ABclonal Biotechnology Co., Ltd.), IκBα mouse mAb (Cat. L35A5, Cell Signaling Technology, Inc.), phospho-IκBα-S32/36 rabbit pAb (Cat. AP0614, ABclonal Biotechnology Co., Ltd.), phospho-NF-κB p105 antibody (Cat. F400, Selleck.), Bcl-2 rabbit mAb (Cat. A19693, ABclonal Biotechnology Co., Ltd.), anti-Bax (Cat. Ab32503, Abcam), Bv421 anti-mouse CD71 (Cat.113813, Biolegend), APC anti-mouse CD34 (Cat.119309, Biolegend), and APC-Cy7 anti-mouse CD45 (557659, BD) antibodies were used.

**TABLE 1:** Basic information of crude drugs in XNX tablets.

Latin name of the crude drug	Full species name(s)	Authorites	Family	Botanical drugs	Processing and extraction of the crude drug	Traditional processing
*Rhodiolae crenulatae radix et rhizoma*	*Rhodiola crenulata*	(Hook. f. et Thoms.) H. Ohba	Crassulaceae	Dried root and rhizome	The drug was extracted twice by reflux with 70% ethanol. For the first extraction, an 8-fold volume of solvent was added and the extraction proceeded for 3 h. For the second extraction, a 6-fold volume of solvent was added and the extraction lasted for 2 h. The combined extracts were filtered and concentrated under reduced pressure.	Cooking
*Lycii fructus*	*Lycium barbarum*	Carolus Linnaeus	Solanaceae	Dried ripe fruit	The drug was decocted twice with water. For the first decoction, a 10-fold volume of water was added and the process lasted for 2 h. For the second decoction, an 8-fold volume of water was added and the process also lasted for 2 h. The decoctions were combined, filtered, and concentrated.	Cooking
*Hippophae fructus*	*Hippophae rhamnoides*	Carolus Linnaeus	Elaeagnaceae	Dried ripe fruit	Fruits were manually sorted to remove moldy or damaged ones. The selected fruits underwent low-temperature physical pressing. The pressed juice was clarified by removing solids (pomace and seeds) through filtration or centrifugation to obtain a clear primary juice. The juice was finally sterilized using pasteurization or instant high-temperature sterilization.	Cooking

### 2.2 Description of the extract and extraction process

XNX Tablets are prepared from extracts of Radix et Rhizoma [*Rhodiola crenulata* (Hook. f. & Thomson) H. Ohba, (Crassulaceae)], Lycii Fructus [*Lycium barbarum* L., (Solanaceae)], and *Hippophae rhamnoides*. The product is approved under registration number Z20090113 and is manufactured in accordance with the standard YBZ02242009, issued by the former China Food and Drug Administration. Basic information regarding the crude drugs used in XNX Tablets is summarized in Table 1. The primary chemical metabolites of the formulation include salidroside, tyrosol, gallic acid, ethyl gallate, p-coumaric acid, caffeic acid, and chlorogenic acid. The production process follows the internally reviewed and approved standard operating procedure SOP-MPP-082 (01), drafted in accordance with the official execution standards.

Using the preparation parameters for 1,000 tablets (0.52 g each) as an example, the formulation requires 1,000 g of *Rhodiola crenulata*, 500 g of *Lycium barbarum*, and 143 g of fresh *Hippophae rhamnoides* pulp. *Rhodiola crenulata* was subjected to reflux extraction twice with 70% ethanol:first with an 8-fold solvent volume for 3 h, followed by a 6-fold solvent volume for 2 h. The combined extracts were concentrated to recover ethanol. *Lycium barbarum* was decocted twice with water, initially with a 10-fold volume for 2 h and subsequently with an 8-fold volume for 2 h. The combined decoctions were then filtered. The fresh *Hippophae rhamnoides* pulp was mixed with the ethanol extract and aqueous decoction, and concentrated to a relative density of 1.05–1.10 (at 50 °C). Corn starch was added to the concentrated mixture, which was then heated, blended, and subjected to spray drying. Subsequently, 60 g of pregelatinized starch, 10 g of crospovidone, and 10 g of sodium starch glycolate were uniformly incorporated. Granulation was performed using 90% ethanol, and the resulting granules were dried at 50 °C–60 °C, passed through a 20-mesh screen, compressed into 1,000 tablets, and finally film-coated.

The resulting crude drug-to-extract ratios are approximately 7.1:1 for *Rhodiola crenulata*, 2.4:1 for *Lycium barbarum*, and 4.8:1 for *Hippophae rhamnoides* pulp (pulp to solids). Each tablet contains approximately 0.14 g of *Rhodiola crenulata* extract, 0.21 g of *Lycium barbarum* extract, 0.03 g of *Hippophae rhamnoides* pulp, and 0.14 g of pharmaceutical excipients, including pregelatinized starch, crospovidone, and sodium starch glycolate.

### 2.3 Animals and modeling

Male KM mice (body weight: 15–16 g, SPF grade) were purchased from Beijing Huafukang Biological Technology. (Production License No. SCXK [Jing] 2019-0008). The animals were housed in the SPF-level Laboratory of IMPLAD, Chinese Academy of Medical Sciences (ethical review number: SLXD-20240604018; License No. SYXK [Jing] 2023-0008). All animal experiments were conducted in accordance with the ARRIVE guidelines and the National Institutes of Health Guidelines for the Care and Use of Laboratory Animals (NIH Publication No. 8023, revised 1978). Following 1 week of acclimation, experimental procedures were initiated.

To establish a model of HAPC induced by HH, mice were placed in a hypobaric chamber and periodically decompressed. The chamber was maintained at a simulated altitude of 5,500 m for 20 h per day, after which it was recompressed to sea level at a rate of 20 m/s. This HH exposure protocol was continued for 1 month.

### 2.4 Experimental groups

A total of 105 KM mice were weight-randomized into seven experimental groups (n = 15 per group): a control group, an HH model group (both receiving normal saline), an XNX low-dose group (XNX-L, 107.65 mg/kg), an XNX medium-dose group (XNX-M, 215.3 mg/kg), an XNX high-dose group (XNX-H, 430.6 mg/kg), an XNX group (430.6 mg/kg), and a positive control group receiving acetazolamide (ACZ, 50.0 mg/kg).

Mice in the control and XNX groups were maintained under normoxic conditions, while the remaining five groups were housed in the HH chamber for 30 days. During this period, the control and HH model groups received normal saline via oral gavage, whereas the other groups were administered the corresponding doses of XNX tablets or ACZ. The gavage volume was adjusted according to body weight at 0.1 mL per 10 g.

### 2.5 Hematological and hemorheological parameters

Two hundred microliters of blood were collected into a 2 mL EDTA anticoagulation tube and gently inverted to ensure proper anticoagulation. After temporary storage at 4 °C, hematological parameters, including white blood cell (WBC) count, RBC count, hemoglobin (HGB), hematocrit (HCT), and platelet (PLT) count, were measured using a blood cell analyzer.

Additionally, 800 μL of blood was collected into a 5 mL heparin anticoagulant tube, inverted for anticoagulation, and temporarily stored at 4 °C. Hemorheological indices were subsequently determined using a Prism LBY-N6C automatic hemorheometer.

### 2.6 Bone marrow cell isolation and flow cytometry

Mice were euthanized via cervical dislocation. The skin of both lower limbs was removed, and the hind limbs were carefully dissected. After removal of attached muscle tissues, the femurs and tibias were isolated. Both ends of the bones were trimmed to expose the red marrow cavity, which was then flushed with 1 mL of PBS. Flushing was repeated 2-3 times. The resulting cell suspension was gently pipetted to dissociate cell aggregates, filtered through a sieve, and collected. The mixture was centrifuged at 300× g for 5 min, and the cell pellet was retained.

The cell density was adjusted to 1 × 10^7^ cells/mL using cell staining buffer. Cells were incubated with the recommended amounts of fluorescently labeled antibodies against CD45, CD34, and CD71 in the dark for 15 min. Subsequently, 1 mL of D Pharm Lyse™ Lysing Buffer was added, and the mixture was incubated in the dark for 5 min. After centrifugation, the supernatant was discarded, and the cells were washed with 1 mL of binding buffer. Finally, the cell pellet was stained with Annexin V as recommended, incubated for 15 min, and analyzed. Flow Cytometry Gating Strategy: Initial gating: Live single cells were identified by excluding cellular debris based on forward scatter (FSC) and side scatter parameters, followed by doublet exclusion using FSC-A versus FSC-H. Identification of target populations: From the singlet gate, two populations were analyzed: Hematopoietic stem/progenitor cells: gated as CD34^+^CD45^+^ cells. Erythroid precursors: gated as CD45^−^CD71^+^ cells. Apoptosis assay of erythroid precursors: CD45^−^CD71^+^ erythroid precursors were further analyzed for apoptosis using Annexin V and propidium iodide (PI) staining. Quadrant gates were applied on the Annexin V versus PI plot to define four subpopulations: Q1 (Annexin V^−^PI^+^): Late apoptotic/necrotic cells. Q2 (Annexin V^+^ PI^+^): Late apoptotic cells. Q3 (Annexin V^+^PI^−^): Early apoptotic cells. Q4 (Annexin V^−^ PI^−^): Viable cells.

### 2.7 Determination of the serum EPO concentration

EPO levels in mouse serum were measured using an EPO assay kit (Cat. SEA028Mu; Cloud-Clone Corp., Wuhan) according to the manufacturer’s instructions.

### 2.8 Cardiac histology

Mouse hearts were fixed in paraformaldehyde, dehydrated in sucrose, and embedded in optimal cutting temperature compound (Cat. G6059; Wuhan Service Bio-Technology Co., Ltd.). Histological analysis was performed using a hematoxylin-eosin (H&E) staining kit (Cat. G1076; Wuhan Service Technology Co., Ltd.) following the manufacturer’s instructions.

### 2.9 Echocardiography

Measurements were performed via a Vevo 1100 miniature ultrasound system (FUJIFILM) with 400 MHz mechanical accessories. The experimental animals were anesthetized with isoflurane throughout the procedure, and the heart rate was maintained at approximately 450–500 beats/minute. The probe was placed parallel to the sternum to obtain a view of the RV, and in B mode, pulmonary artery (PA) images were acquired.

### 2.10 Measurement of inflammatory cytokine levels

Serum concentrations of tumor necrosis factor-α (TNF-α), interleukin-1β (IL-1β), and interleukin-6 (IL-6) were quantified via enzyme-linked immunosorbent assay (ELISA) kits (TNF-α, Cat# 920; IL-1β, Cat# 3748; IL-6, Cat# 4355; Jiangsu Enzyme Immunity Industry). Perform according to the instructions.

### 2.11 Assessment of oxidative stress status

Myocardial oxidative stress status was evaluated by measuring malondialdehyde (MDA) content and the activities of total antioxidant capacity (T-AOC, Cat# A015-2-1), catalase (CAT, Cat# A007-1-1), and superoxide dismutase (SOD, Cat# A001-3-2) via specific kits (Nanjing Jiancheng Bioengineering Institute) according to the provided instructions.

### 2.12 Network analysis

The structures and identifiers of the 177 metabolites identified through UPLC-Q-TOF-MS analysis of XNX were retrieved from the PubChem database and ChemDraw. Meanwhile, bioactive metabolites in XNX were collected from the TCMSP, HERB and SymMap databases. To mitigate false positive predictions, screening was performed based on oral bioavailability (OB) ≥ 30% and drug-likeness (DL) ≥ 0.18. Given the absence of *in vivo* pharmacokinetic data specific to the formulation in this study, the candidate metabolites represent a theoretical chemical space rather than confirmed bioactive metabolites. The chemical metabolites were obtained with SMILES numbers on the pubchem website and analyzed through the SwissStargetPrediction, SwissADME and ChemFH databases to eliminate the interfering compounds and implausible leads metabolites and their targets. This screening process allows us to reduce the weight of high-risk candidates and reduce the systematic over-prioritization of confounding molecules. It should be noted that as a virtual screening tool, the prediction results of network pharmacology are limited by database bias and algorithms, and there is the limitation of homogenization pointed out by Diao et al. (2026). Therefore, the network pharmacological analysis in this study was used only for preliminary screening and auxiliary hypothesis generation, not as independent evidence of pharmacological activity. All predicted pathways and targets need to be validated and confirmed by independent experiments (Western blotting).

Disease-related targets for high-altitude polycythemia (HAPC) were collected by querying the OMIM and GeneCards databases using the keyword “high altitude polycythemia.” Overlaps between XNX and disease targets were obtained and visualized using an online Venn diagram tool. The intersecting targets were defined as the core targets of XNX against HAPC and were used for further analysis. The intersection of XNX and HAPC targets was defined as the potential therapeutic target genes of XNX for HAPC. Protein‒protein interaction (PPI) network analysis was performed for the identified human targets via the STRING database (v12.0). The resulting network was imported into Cytoscape (v3.10.0) for visualization and topological analysis to identify core targets. Due to the absence of transcriptomic or metabolomic data specific to the disease model, target prioritization was further refined through literature-based pathway enrichment rather than relying solely on topological metrics. Centrality measures—including degree centrality, betweenness centrality (to identify network bottlenecks), and eigenvector centrality (to weight influential nodes)—were integrated to better prioritize targets with pathological specificity as candidate genes for subsequent studies. The enrichment of targets was then carried out via the DAVID database (2021) for Gene Ontology (GO) terms and Kyoto Encyclopedia of Genes and Genomes (KEGG) pathways, with the results visualized on the bioinformatics online platform (https://www.bioinformatics.com.cn/).

### 2.13 Western blotting

Equal amounts of protein were separated by SDS‒PAGE and transferred to PVDF membranes. The membranes were blocked in skim milk for 2 h and then incubated overnight at 4 °C with primary antibodies, including MAPK rabbit pAb (1:1000), p38 MAPK rabbit mAb (1:2000), JNK1/2/3 rabbit mAb (1:1000), phospho-JNK rabbit mAb (1:1000), ERK1/2 rabbit mAb (1:2000), phospho-ERK1/2 rabbit mAb (1:2000), AKT rabbit antibody (1:1000), phospho-AKT (Ser473) monoclonal antibody (1:2000), IκBα mouse mAb (1:1000), phospho-IKBα-S32/36 rabbit pAb (1:500), NF-κB p65 antibody (1:1000), phospho-NF-κB p105 (Ser932) antibody (1:1000), Bcl-2 rabbit mAb (1:1000), and anti-Bax antibody (1:1000). After being washed with TBST, the membranes were incubated with secondary antibodies at room temperature for 2 h, followed by chemiluminescent detection.

### 2.14 Statistical analysis

The data are presented as the means ± standard errors of the means (SEMs) and were analyzed via GraphPad Prism 8.0. Data normality was assessed via the Shapiro–Wilk test.

For normally distributed data, comparisons between two groups were performed with a two-tailed unpaired t-test. One-way or two-way ANOVA was used for comparisons among three or more groups, followed by Tukey’s multiple comparisons test. For nonnormally distributed data, the Kruskal‒Wallis test was used. A *p* value < 0.05 was considered statistically significant.

## 3 Results

### 3.1 Identification of XNX metabolites

**FIGURE 1:**
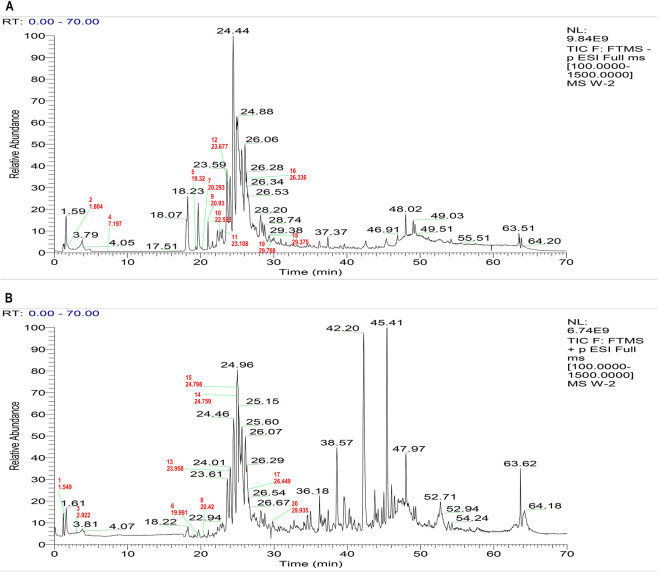
Identification of XNX tablets via UPLC‒Q‒TOF‒MS. **(A)** Negative ion mode and **(B)** positive ion mode.

The UPLC diagrams of XNX are shown in Figure 1.

The chemical metabolites of XNX tablets were analyzed using UPLC-Q-TOF-MS, leading to the identification of 177 distinct chemical structures (Supplementary File 1). Among these metabolites, the most abundant were flavonoids and their glycosides, comprising approximately 50 metabolites, including quercetin, kaempferol, rutin, astragalin, glycitein, pelargonidin, and (+)-catechin. Phenolic acids and their derivatives accounted for about 30 metabolites, such as gallic acid, ethyl gallate, caffeic acid, ellagic acid, neochlorogenic acid, cinnamic acid, and ferulic acid. Organic acids represented roughly 20 metabolites, including citric acid and methyl linoleate. Coumarins and lignans were also present, such as esculetin and 7-hydroxycoumarin. Terpenoids identified included β-sitosterol, stigmasterol, and other related metabolites. Alkaloids comprised berberine, nicotinic acid, betaine (a quaternary ammonium alkaloid), and atropine. Additional metabolites included phenylpropanoids and simple aromatic metabolites, such as salidroside. We selected the main 20 metabolites defined in the pharmacopoeia for presentation (Table 2).

**TABLE 2:** Analysis and identification of the chemical metabolites in XNX tablets.

No	RT (min)	Chemical name	Formula	Annot. delta mass [ppm]	m/z	Reference
1	1.549	Betaine	C_5_H_11_NO_2_	1.25	118.0864	[M + H] + 1
2	1.804	Citric acid	C_6_H_8_O_7_	−0.54	191.0196	[M − H] −1
3	2.922	Nicotinic acid	C_6_H_5_NO_2_	2.06	124.0396	[M + H] + 1
4	7.197	Gallic acid	C_7_H_6_O_5_	−1.23	169.014	[M − H] − 1
5	19.352	Salidroside	C_14_H_20_O_7_	−0.96	345.1188	[M + FA − H] − 1
6	19.991	Citral	C_10_H_16_O	1.43	153.1276	[M + H] + 1
7	20.293	Neochlorogenic acid	C_16_H_18_O_9_	−0.88	353.0875	[M − H] − 1
8	20.42	Trans-Cinnamaldehyde	C_9_H_8_O	1.25	133.065	[M + H] + 1
9	20.93	Esculetin	C_9_H_6_O_4_	−0.84	177.0191	[M − H] − 1
10	22.533	Ethyl gallate	C_9_H_10_O_5_	−1.09	197.0453	[M − H] − 1
11	23.108	Ferulic acid	C_10_H_10_O_4_	−0.96	193.0504	[M − H] − 1
12	23.677	Astragalin	C_21_H_20_O_11_	−0.8	447.0929	[M − H] − 1
13	23.958	Myricetin	C_15_H_10_O_8_	0.92	319.0451	[M + H] + 1
14	24.759	Eugenol	C_10_H_12_O_2_	1.84	165.0913	[M + H] + 1
15	24.798	Rutin	C_27_H_30_O_16_	0.19	611.1614	[M + H] + 1
16	26.336	Quercetin	C_15_H_10_O_7_	−0.62	301.0351	[M − H] − 1
17	26.449	Berberine	C_20_H_17_NO_4_	0.77	336.1233	[M + H] + 1
18	29.375	Kaempferol	C_15_H_10_O_6_	−0.52	285.0402	[M − H] − 1
19	29.768	Isorhamnetin	C_16_H_12_O_7_	0.36	315.051	[M − H] − 1
20	29.935	Cinnamic acid	C_9_H_8_O_2_	1.76	149.06	[M + H] + 1

### 3.2 Effects of XNX on HH-induced hematological parameters in HAPC model mice

**FIGURE 2:**
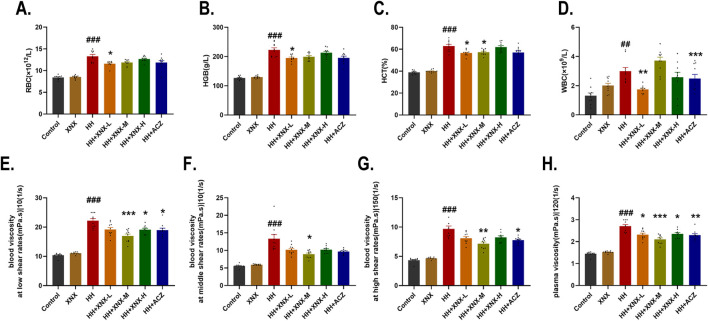
Impact of XNX on hematological indices in HPAC mice. **(A)** RBC, n = 9–10. **(B)** HGB, n = 9–10. **(C)** HCT, n = 9–10. **(D)** WBC, n = 9–10. **(E)** Low shear whole blood viscosity, n = 9–10. **(F)** Medium shear whole blood viscosity, n = 9–10. **(G)** High shear whole blood viscosity, n = 9–10. **(H)** Plasma viscosity, n = 9–10. ^###^ *p* < 0.001, ^##^ *p* < 0.01, ^#^ *p* < 0.05, vs. the control group; ***p < 0.001, **p < 0.01, *p < 0.05 vs. the HH group. Control: normoxia control; XNX: normoxia Xinnaoxin; HH: hypobaric hypoxia; XNX-L: XNX low-dose; XNX-M: XNX medium-dose; XNX-H: XNX high-dose; ACZ: acetazolamid.

CBC analysis was performed on mouse whole blood to assess systemic hematological profiles across the experimental groups. As shown in Figures 2A–D, HH exposure induced significant increases in RBC count, HCT, HGB levels, and WBC count, consistent with the hematological characteristics of HAPC. Treatment with a low dose of XNX effectively reversed these HH-induced elevations.

Excessive erythrocytosis can increase blood viscosity and impair circulation. Accordingly, hemorheological parameters were measured. The HH group exhibited significantly elevated whole blood viscosity at low, medium, and high shear rates, as well as increased plasma viscosity, compared with normoxic controls. In contrast, treatment with XNX or ACZ markedly reduced these parameters relative to the HH group (Figures 2E–H).

### 3.3 Modulation of erythropoiesis by XNX in a murine model of HH-induced HAPC

**FIGURE 3:**
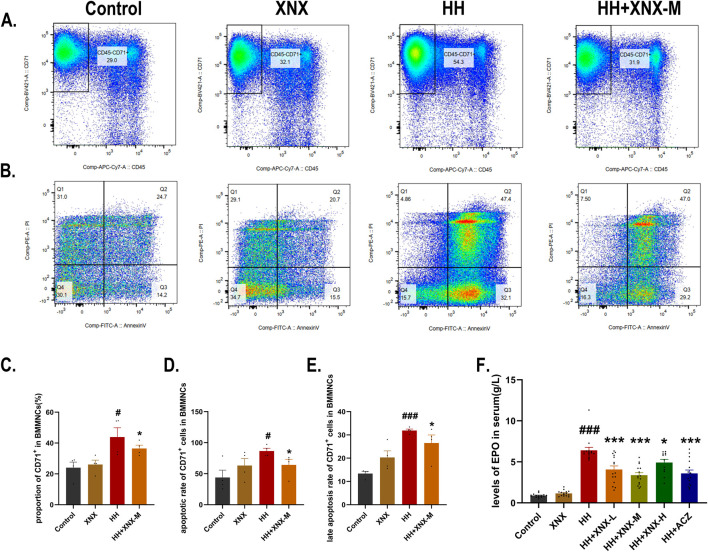
Effect of XNX on erythropoiesis in mice with HH-induced polycythemia. **(A)** CD71^+^ erythroblast proportions in the bone marrow. **(B)** Apoptosis rate of bone marrow CD71^+^ erythroblasts. **(C)** Quantitative analysis of the proportion of bone marrow CD71^+^ erythroblasts, n = 4. **(D, E)** Analysis of the (late) apoptosis rate of bone marrow CD71^+^ erythroblasts, n = 4. **(F)** EPO content in the serum, n = 12–15. ^###^ *p* < 0.001, ^##^ *p* < 0.01, ^#^ *p* < 0.05, vs. the control group; ***p < 0.001, **p < 0.01, *p < 0.05 vs. the HH group. Control: normoxia control; XNX: normoxia Xinnaoxin; HH: hypobaric hypoxia; XNX-L: XNX low-dose; XNX-M: XNX medium-dose; XNX-H: XNX high-dose; ACZ: acetazolamide.

Maintaining a dynamic balance between blood cell production and apoptosis is essential for normal hematopoiesis. CD71, a transferrin receptor predominantly expressed on erythroid precursor cells, serves as a key marker of erythropoietic activity. Consistent with previous reports, our data demonstrate that HH exposure exerts a dual effect on bone marrow: CD71^+^ cells proliferate abnormally while simultaneously exhibiting increased apoptosis. Notably, we provide the first evidence that XNX treatment effectively attenuates both the HH-induced expansion of the CD71^+^ cell population and the associated apoptosis rate (Figures 3A–E).

EPO, a glycoprotein hormone, plays a critical role in regulating the proliferation and maturation of erythroid cells. Hypoxia induces a rise in plasma EPO levels, promoting increased RBC production. As shown in Figure 3F, serum EPO levels were significantly elevated in HH-exposed mice compared with normoxic controls. Therapeutic administration of XNX or ACZ substantially normalized EPO concentrations, restoring them toward baseline levels.

### 3.4 Cardioprotective effects of XNX against HH-induced HAPC

As altitude increases, hemodynamic load rises to meet the oxygen demands of the myocardium. However, this compensatory response is accompanied by impaired cardiac pump function, prolonged systolic intervals, and increased blood viscosity. These changes promote blood stasis, elevate microcirculatory resistance, reduce oxygen delivery to vital organs, and ultimately contribute to cardiac injury.

**FIGURE 4:**
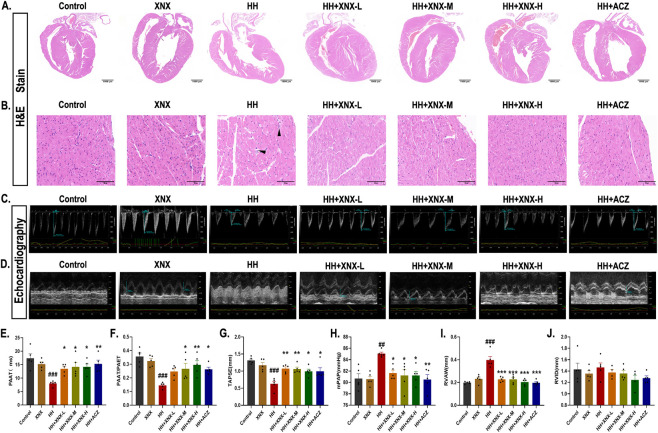
Effects of XNX on HH-induced changes in heart morphology and RV function in mice. **(A)** Representative H&E staining image of a mouse heart; n = 4 (150 ×, 1000 μm; 400 ×, 50 μm). **(B)** Representative H&E-stained graph of mouse heart regional RVs; n = 4 (500 ×, 100 μm). **(C, D)** Representative ultrasound image of mouse RV function; n = 5. **(E–H)** Statistical results of mouse PAAT, PAAT/PAET, TAPSE and mPAP, n = 5. **(I, J)** Statistical results of mouseRVAW and RVID, n = 5. ^###^ *p* < 0.001, ^##^ *p* < 0.01, ^#^ *p* < 0.05, vs. the control group; ***p < 0.001, **p < 0.01, *p < 0.05 vs. the HH group. Control: normoxia control; XNX: normoxia Xinnaoxin; HH: hypobaric hypoxia; XNX-L: XNX low-dose; XNX-M: XNX medium-dose; XNX-H: XNX high-dose; ACZ: acetazolamide.

Histopathological analysis using H&E staining (Figures 4A,B) revealed that, under normoxic conditions, cardiomyocytes were uniformly distributed, myofiber structures were clearly defined and orderly, the extracellular matrix was minimal, and nuclear density (blue) was moderate. In contrast, HH exposure resulted in marked thickening of myocardial fibers, widened interstitial spaces, disorganized tissue architecture, and prominent inflammatory cell infiltration. Treatment with XNX tablets or ACZ ameliorated these pathological changes, reducing fiber thickness, narrowing interstitial spaces, and diminishing or eliminating inflammatory infiltration.

Echocardiographic assessment of RV morphology, including RV anterior wall (RVAW) thickness and RV internal diameter (RVID), is presented in Figures 4I,J. HH-exposed mice exhibited significant RVAW thickening compared with normoxic controls. Both XNX and ACZ markedly attenuated this hypertrophic response. Although RVID showed an increasing trend under HH conditions and a decrease following drug administration, these changes did not reach statistical significance.

Parameters of RV systolic function, including tricuspid annular plane systolic excursion (TAPSE), pulmonary artery acceleration time (PAAT), PAAT/pulmonary artery ejection time (PAET) ratio, and estimated mean pulmonary arterial pressure (mPAP), are shown in Figures 4C–H. HH exposure significantly decreased PAAT, PAAT/PAET, and TAPSE, while increasing mPAP relative to controls. Administration of XNX or ACZ significantly improved RV systolic function by increasing PAAT, PAAT/PAET, and TAPSE and reducing mPAP compared with the HH group.

### 3.5 Results of network analysis of XNX-treated HH-induced HAPC mice

UPLC-Q-TOF-MS/MS chemical analysis identified 177 metabolites present in Xinnaoxin tablets (Supplementary File 1). This step ensures that our starting point is the actual chemical composition of the herbal product, rather than a pure database-derived list. We supplemented the metabolite lists and target genes of the three medicinal materials from the TCMSP and HERB databases, which were pre-filtered using conventional parameters (OB ≥ 30%, DL ≥ 0.18) and eliminated target-free metabolites (Supplementary File 2). Intersecting 177 metabolites in Supplementary File 1 with metabolites in Supplementary Document 2 yielded 18 metabolites (Supplementary File 3).

Through this comprehensive process, we obtained 18 metabolites that were used for target prediction. We acknowledge and clearly state in the manuscript: Because *in vivo* pharmacokinetic data for specific formulations are not available, these 18 metabolites represent theoretical chemical space and not confirmed bioavailable metabolites. This is an explicit limitation, and we classify our approach as a “minimally feasible” implementation that follows the phased roadmap proposed by Diao et al. (2026). In addition, according to the requirements of Diao et al. (2026), we obtained 18 chemical metabolites with SMILES numbers on the pubchem website, entered them into the SwissADME website for filtering, and obtained Supplementary File 4 and Supplementary File 5. The results showed that the 18 chemical metabolites screened above were eligible for analysis. Further, we analyzed SMILES numbers of 18 chemical metabolites using the ChemFH database. Screening Mode is able to screen a molecular dataset and detect potential frequent hitters, thus improving the credibility of experimental results and decreasing unnecessary costs. According to the screening results (Supplementary File 6), we eliminated the potential frequent hitters such as 7-O-Methylluteolin-6-C-beta-glucoside_qt, quercetin and pelargonidin and their targets, and obtained the remaining 15 chemical metabolites and their target genes (Supplementary File 7). This screening process allows us to reduce the weight of high-risk candidates and reduce systematic over-prioritization of confounding molecules.

**FIGURE 5:**
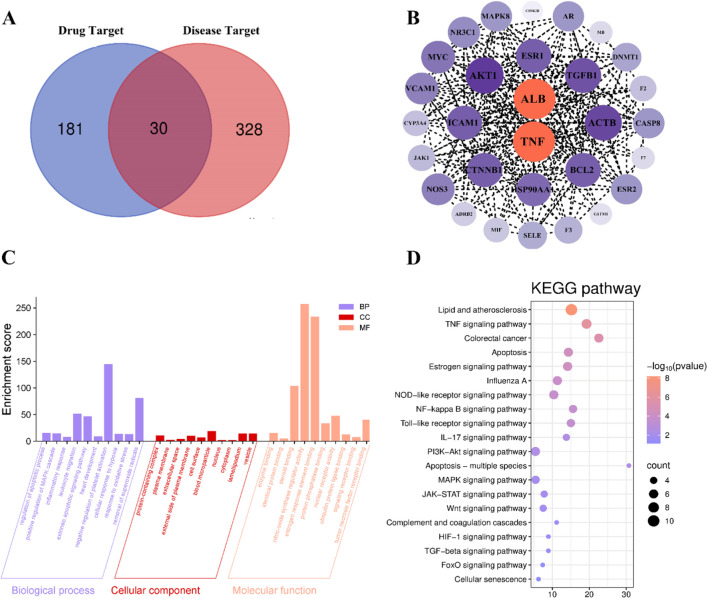
An integrative network analysis approach to elucidate the therapeutic mechanisms of XNX against HAPC. **(A)** Venn diagram of the shared targets between XNX and HAPC. **(B)** PPI network of the common targets. Node size and color intensity represent the degree of connectivity. **(C)** GO functional enrichment analysis. **(D)** KEGG pathway enrichment analysis.

A total of 358 HAPC-related target genes were collected from the GeneCards and OMIM databases (Supplementary File 8). A Venn diagram (Figure 5A) revealed that 30 common targets were identified as candidate therapeutic targets by intersecting the targets of XNX and HAPC (Supplementary File 9). These 30 common targets were analyzed using the STRING platform to map potential protein–protein interactions (Figure 5B). Owing to the absence of transcriptomic or metabolomic data specific to the disease model, target prioritization was further refined through literature-based pathway enrichment rather than relying solely on topological metrics. In the current PPI network, we considered not only simple metrics such as degree centrality but also betweenness centrality (to identify network bottlenecks) and eigenvector centrality (to weight influential nodes), thereby better prioritizing targets with pathological specificity. Using the approach described above, BCL2, TNF, AKT1, TGFB1, MAPK8, ICAM1, and VCAM1 were selected as candidate therapeutic targets (Supplementary File 10). Not only based on chemical popularity, but on integration with protein-protein interaction network topologies and subsequent *in vitro* experimental validation (ELISA and Western blot). We also note that our use of *in vitro* experimental validation was a direct response to the criticism made by Diao et al. (2026). That is, the calculated prediction must be followed by rigorous testing to distinguish between true pharmacological signals and artifacts.

To elucidate the multifaceted biological functions of XNX and its mechanisms for ameliorating HAPC, the 50 common targets were imported into the DAVID database for GO and KEGG enrichment analyses. A GO plot was generated to display the corresponding enrichment results, including the top 10 biological process (BP) terms. Regarding BPs, the intersecting targets were primarily enriched in processes related to the inflammatory response (e.g., inflammatory response, leukocyte migration), oxidative stress and reactive oxygen species metabolism (positive regulation of MAPK cascade, cellular response to hypoxia, response to oxidative stress, removal of superoxide radicals), regulation of apoptosis (regulation of apoptotic process), and negative regulation of platelet activation (Figure 5C). As shown in Figure 5D, the top 20 KEGG pathways associated with HAPC included inflammatory responses (TNF, NF-κB, MAPK, JAK, and TGF-β signaling pathways), cell proliferation and survival (PI3K-Akt and HIF-1 signaling pathways), apoptosis, platelet activation, and hypertrophic cardiomyopathy. Therefore, in this study, we selected the predicted pathways related to inflammation, oxidative stress and reactive oxygen species metabolism, and cell proliferation and apoptosis to investigate the potential mechanisms by which XNX treats HAPC.

It is important to note that, in line with the phased roadmap proposed in recent methodological guidelines, the present study represents a “minimum viable” implementation (Diao et al., 2026), constrained by the absence of formulation-specific pharmacokinetic data and multi-omics profiling. Accordingly, our findings should be interpreted as hypothesis-generating rather than mechanistic validation. To test these predictions, we next performed ELISA and Western blot analyses, which confirmed that XNX treatment significantly modulated markers of oxidative stress, inflammation, and apoptosis in cardiomyocytes, thereby providing experimental evidence for the pathways suggested by our network analysis.

### 3.6 XNX ameliorate inflammation and oxidative stress in the myocardium of HAPC mice

**FIGURE 6:**
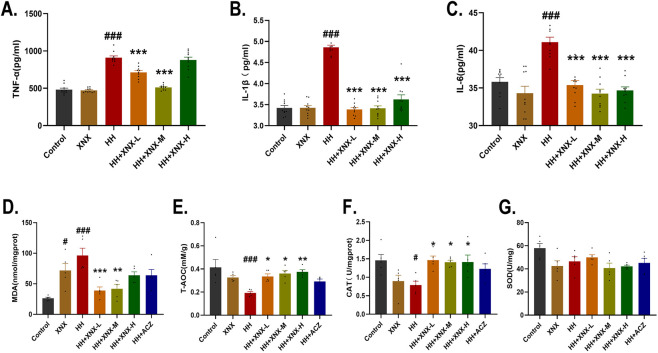
Effects of XNX on HH-induced inflammation and oxidative stress indices in cardiomyocytes from mice. Contents of the inflammatory factors **(A)**, TNF-α **(B)**, IL-1β **(C)** and IL-6 in the serum, n = 10. **(D)** MDA content in cardiac tissue; n = 5. **(E–G)** T-AOC and CAT and SOD activities in cardiac tissue; n = 5. ^###^ *p* < 0.001, ^##^ *p* < 0.01, ^#^ *p* < 0.05, vs. the control group; ***p < 0.001, **p < 0.01, *p < 0.05 vs. the HH group. Control: normoxia control; XNX: normoxia Xinnaoxin; HH: hypobaric hypoxia; XNX-L: XNX low-dose; XNX-M: XNX medium-dose; XNX-H: XNX high-dose; ACZ: acetazolamide.

TNF-α, IL-1β, and IL-6 are key proinflammatory cytokines that play critical roles in the inflammatory response. Analysis of serum inflammatory factor levels revealed that HH exposure significantly increased the concentrations of these cytokines compared with the control group. Treatment with XNX markedly reduced serum levels of TNF-α, IL-1β, and IL-6 in HH-exposed mice. Notably, while the HH + XNX-H group showed a modest reduction in TNF-α compared with the HH group, this difference did not reach statistical significance (Figures 6A–C).

Malondialdehyde (MDA) is a recognized biomarker of oxidative stress that reflects the severity of cellular damage. HH exposure significantly elevated MDA levels in cardiac homogenates compared with normoxic controls (Figure 6D). Supplementation with low- or medium-dose XNX significantly reduced cardiac MDA levels in HH mice. Total antioxidant capacity (T-AOC) reflects overall antioxidant potential, while superoxide dismutase (SOD) and catalase (CAT) are key enzymatic antioxidants. HH exposure significantly decreased T-AOC and CAT activity in cardiac tissue, which were effectively restored by XNX treatment (Figures 6E,F). Although cardiac SOD activity showed a decreasing trend under HH and an increasing trend with low-dose XNX, these changes were not statistically significant (Figure 6G).

Notably, treatment with XNX under normoxic conditions produced a significant effect only on MDA levels, without significantly altering other markers of oxidative stress or inflammation.

### 3.7 XNX can effectively reverse the significant reduction of pAKT/AKT and the increase of pNF-κB/NF-κB and pMAPK/MAPK in the heart of HH mice

**FIGURE 7:**
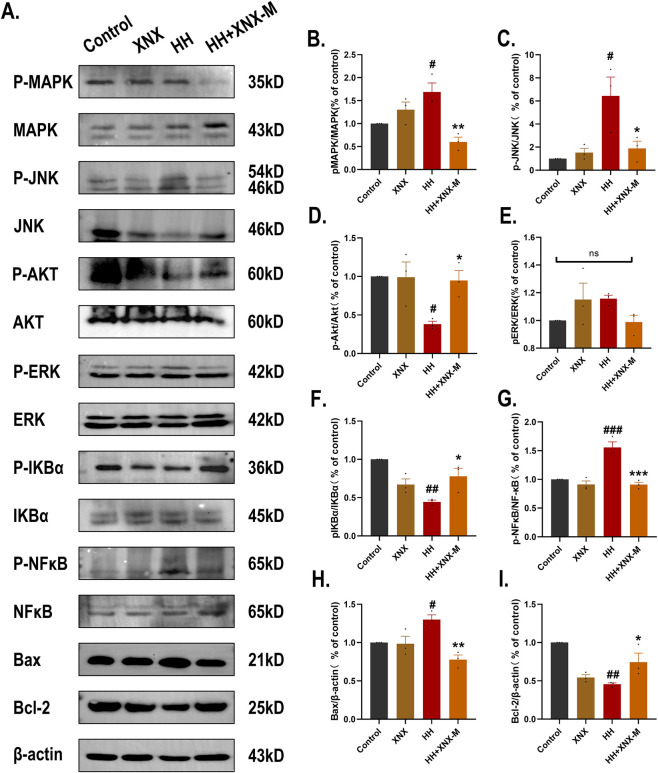
Effect of XNX on the expression of MAPK and NF-κB signaling pathway-related proteins in the RVs of mice with HH heart injury. **(A)** Representative protein bands. Quantitative statistical results of **(B)** p-MAPK/MAPK, **(C)** p-JNK/JNK, **(D)** p-Akt/Akt, **(E)** p-ERK/ERK, **(F)** p-IKBα/IKBα, **(G)** p-NF-κB/NF-κB, **(H)** Bax/β-actin, and **(I)** Bcl-2/β-actin, n = 3. ^###^ *p* < 0.001, ^##^ *p* < 0.01, ^#^ *p* < 0.05, vs. the control group; ***p < 0.001, **p < 0.01, *p < 0.05 vs. the HH group. Control: normoxia control; XNX: normoxia Xinnaoxin; HH: hypobaric hypoxia; XNX-L: XNX low-dose; XNX-M: XNX medium-dose; XNX-H: XNX high-dose; ACZ: acetazolamide.

The MAPK and NF-κB signaling pathways, along with apoptosis-related signaling, play central roles in the pathogenesis of HH-induced cardiac injury. To elucidate the molecular mechanisms by which XNX tablets confer cardioprotection, Western blotting was employed to examine the modulation of these pathways. HH exposure significantly increased the phosphorylation of key proteins in the NF-κB pathway (IKBα and NF-κB) and the MAPK pathway (MAPK and JNK) in mouse cardiac tissue compared with normoxic controls. Conversely, HH markedly decreased phosphorylation of AKT in the PI3K/AKT survival pathway. Treatment with XNX effectively reversed these HH-induced alterations, suppressing overactivation of NF-κB and MAPK signaling while restoring AKT phosphorylation. Notably, pERK/ERK levels within the MAPK pathway remained essentially unchanged under HH conditions. Additionally, HH exposure significantly upregulated the pro-apoptotic protein BCL2-associated X protein (Bax) and suppressed the anti-apoptotic protein B-cell lymphoma 2 (Bcl-2) in cardiac tissue. XNX administration significantly mitigated these changes, restoring the balance of apoptosis-related proteins toward a cardioprotective profile (Figure 7).

## 4 Discussion

The detrimental health effects of HH environments have long been a major concern. Prolonged residence at high altitude is reported to cause abnormal erythrocytosis, which is frequently associated with heart failure (Salmon and Satchell, 2012). Moreover, high-altitude illnesses, such as HAPC, pulmonary hypertension, and RV hypertrophy, are closely linked to oxidative stress (Jefferson et al., 2004; Lüneburg et al., 2016; Sarada et al., 2008; Zhang et al., 2021). HH-induced oxidative stress can disrupt endoplasmic reticulum homeostasis, subsequently triggering inflammatory responses (Marciniak and Ron, 2006). Therefore, elucidating the mechanisms underlying HH-induced HAPC and associated cardiac injury is critical for effective prevention and treatment. XNX tablets, a TMC formulation validated by clinical and preclinical studies, enhance tissue tolerance to hypoxia. They have been shown to differentially improve hypoxia tolerance in cardiac, pulmonary, hepatic, and cerebral tissues (Bernatoniene et al., 2023; Gerbarg and Brown, 2016; Purushothaman et al., 2008; Wu et al., 2025; Xiong et al., 2021; Zheng et al., 2019), exerting protective effects through improved adaptability to hypoxic stress. However, the precise mechanisms underlying its anti-inflammatory and antioxidant effects in the prevention and treatment of HAPC remain unclear. This study aims to systematically elucidate the multi-target synergistic mechanisms of XNX and provide innovative insights for developing therapeutic strategies against altitude-related cardiovascular diseases.

Mature RBCs are the primary carriers of oxygen in the body and are highly sensitive to hypoxia. The classic physiological response to systemic hypoxia is enhanced erythropoiesis, reflected by significant increases in RBC count, HGB concentration, and HCT. While this adaptation aims to improve oxygen delivery, it is a “double-edged sword,” as excessive erythrocytosis can elevate blood viscosity, impair microcirculation, and increase the risk of thrombosis, which has been associated with myocardial infarction (León-Velarde et al., 2005; Penaloza and Arias-Stella, 2007; Vilafuerte and Corante, 2016). In this study, we confirmed that HH exposure induces compensatory increases in hematological parameters, including RBCs, WBCs, and PLTs. More importantly, we demonstrated for the first time that XNX Tablets can comprehensively and significantly reverse these HH-induced hematological and hemorheological abnormalities (Figure 2). This effect extends beyond simple suppression of compensatory responses, suggesting that XNX may help the body achieve a more balanced and adaptive state under hypoxic conditions. These findings reveal the potential of XNX Tablets for improving HH-induced HAPC.

The mechanism by which XNX Tablets attenuate HH-induced erythrocytosis was investigated through measurement of serum EPO levels and bone marrow flow cytometry. Previous studies have suggested that HAPC may be associated with imbalances in the proliferation and apoptosis of bone marrow erythroblasts (Ma et al., 2019; Wang et al., 2022; Zhao et al., 2017). To explore the mechanism by which XNX improves HH-induced polycythemia, we focused on core regulatory pathways of erythropoiesis. EPO plays a crucial role in the generation and maturation of RBC in the bone marrow (Marzo et al., 2008; Ogunshola et al., 2006). Under hypoxic conditions, hypoxia-inducible factors are upregulated (Su et al., 2015; Xie et al., 2016), promoting transcription of target genes such as EPO(Lappin and Lee, 2019; Liu et al., 2018), and accelerating erythrocyte proliferation and maturation. Our study provides novel evidence that XNX specifically targets erythroid precursor cells. Flow cytometry analysis revealed that XNX treatment significantly reduced the proportion of CD71^+^ early erythroid progenitors in the bone marrow. Because CD71 (transferrin receptor) is a marker of active erythroid proliferation, its reduction indicates that XNX inhibits hypoxia-driven excessive and disordered erythroid hyperplasia (Figure 3). Importantly, we further analyzed apoptosis within this cell population. While Harada et al. reported that erythroblast apoptosis increases under acute hypoxia but decreases under chronic hypoxia (Harada et al., 2015), our findings demonstrate a significant increase in erythroblast apoptosis under chronic HH exposure compared with normoxic controls (Figure 3). This observation aligns with the work of El Hoss et al., who showed that erythroblasts cultured under hypoxic conditions can undergo morphological sickling, resulting in ineffective terminal erythroid differentiation and elevated apoptosis, a process termed “ineffective erythropoiesis.” (Hoss et al., 2021). Increased ineffective erythropoiesis can contribute to anemia. Together, these results suggest that chronic hypoxia induces a pathological state of “high output yet low efficiency” in bone marrow erythropoiesis. Our study is the first to link the therapeutic effect of XNX to the emerging concept of ineffective erythropoiesis. Following XNX treatment, the aberrant apoptotic tendency of bone marrow erythroid precursor cells was significantly ameliorated, indicating a restoration of more balanced and effective erythropoiesis.

Taken together, XNX does not merely suppress erythropoiesis in a broad or nonspecific manner. Rather, it exerts dual, innovative effects. On one hand, it moderately attenuates the hypoxic stimulus at its source by downregulating EPO. On the other hand, and more novelly, it appears to improve the bone marrow microenvironment or the intrinsic state of erythroid progenitors, thereby promoting more efficient and mature erythroid differentiation. This dual action reduces the production and apoptosis of “ineffective erythrocytes,” decreasing RBC quantity while simultaneously enhancing the overall quality and efficiency of erythropoiesis. These findings suggest a potential new mechanism by which XNX treats HAPC, one that extends beyond simple EPO inhibition, and provide novel experimental evidence for the prevention and treatment of high-altitude diseases from the perspective of correcting ineffective erythropoiesis. The precise molecular regulatory network underlying these effects warrants further investigation in future studies.

Under HH conditions, an imbalance between ROS production and antioxidant defenses induces oxidative stress, which damages cellular microstructures and ultimately leads to cell injury (Bakonyi and Radak, 2004; Liu et al., 2020; Dosek et al., 2007). MDA, a terminal product of ROS-mediated lipid peroxidation, is widely used as a biomarker to quantify oxidative stress. Moreover, HH-induced oxidative stress can impair endoplasmic reticulum function (Amodio et al., 2018; Pluquet et al., 2015), resulting in the accumulation of unfolded or misfolded proteins, which further trigger and amplify inflammatory responses (Marciniak and Ron, 2006; Julian et al., 2011). Oxidative stress and inflammation are closely interrelated and mutually reinforcing. Proinflammatory cytokines can promote ROS generation, which in turn activates proinflammatory signaling pathways, such as NF-κB, creating a self-amplifying vicious cycle that exacerbates cellular injury. Consistent with this pathological network, HH-exposed mice in our study exhibited elevated MDA levels in myocardial tissue, reduced activity of key antioxidant enzymes (SOD, CAT) and T-AOC, and increased serum levels of proinflammatory cytokines (TNF-α, IL-1β, IL-6) (Figure 6).

A novel finding of this study is that XNX synergistically regulates the oxidative stress-inflammation network. Our results demonstrate that XNX intervention not only effectively reduces cardiac MDA levels and enhances the activity of antioxidant enzymes (SOD, CAT) and T-AOC, but also concurrently suppresses the release of proinflammatory cytokines, including TNF-α, IL-1β, and IL-6 (Figure 6). These findings suggest that XNX does not act on oxidative stress or inflammation in isolation, but instead may exert a coordinated protective effect by targeting upstream co-regulatory nodes and disrupting the self-amplifying vicious cycle between oxidative stress and inflammation.

Of particular interest is the “state-dependent” nature of XNX’s effects. Under normal physiological conditions, XNX induced only minor fluctuations in cardiac MDA levels and did not alter other oxidative or inflammatory markers, nor did it produce signs of toxicity, indicating minimal interference with normal metabolism and good safety. In contrast, under the pathological context of HAPC, XNX exerted a pronounced “corrective” effect, significantly improving both oxidative stress and inflammatory status. This selective activity suggests that XNX preferentially targets dysregulated signaling pathways in pathological conditions, rather than broadly suppressing physiological processes, providing valuable insight into its precise therapeutic properties.

Both oxidative stress and inflammation play central roles in the pathogenesis of cardiac hypertrophy and heart failure (Dewachter and Dewachter, 2018; Hingtgen et al., 2006; Wróbel-Nowicka et al., 2024), with the RV being particularly susceptible to oxidative injury compared with the left ventricle (Borchi et al., 2010; Shults et al., 2018). Previous studies have demonstrated markedly elevated inflammation and oxidative stress in animal models of HH-induced RV hypertrophy and failure (Pena et al., 2020a; Pena et al., 2020b). As described earlier, prolonged hypoxic exposure increases blood viscosity and microcirculatory resistance, resulting in chronic oxygen insufficiency in cardiomyocytes and subsequent myocardial damage (González et al., 2005). A key consequence of sustained HH is the development of pulmonary hypertension (Sommer et al., 2008; Neupane and Swenson, 2017), which frequently leads to RV hypertrophy and, ultimately, heart failure or death (Penaloza and Arias-Stella, 2007). Using multimodal evaluation, including small animal echocardiography and histopathological assessment via H&E staining, we confirmed that the antioxidant and anti-inflammatory effects of XNX translated into tangible cardioprotective outcomes. XNX treatment significantly alleviated HH-induced inflammatory cell infiltration in myocardial tissue (Figures 4A,B) and effectively improved RV systolic function parameters (Figures 4C–J). These findings demonstrate, at both functional and morphological levels, that XNX protects the heart from progression to terminal injury by modulating the core pathological network of oxidative stress and inflammation.

Previous clinical and experimental evidence has shown that the cardioprotective effects of XNX tablets and their principal herbal metabolites are mediated, at least in part, through inhibition of oxidative stress and apoptosis in cardiomyocytes. Interestingly, prior research indicates that HH similarly induces oxidative stress in the brain and activates the Ras-MAPK pathway, exacerbating neural damage, which suggests a shared, tissue-spanning mechanism of action (Chen et al., 2021). In this study, we sought to explore the potential mechanisms underlying the therapeutic effects of XNX. To generate testable hypotheses, we first employed an integrated approach combining UPLC‒MS analysis with network analysis. This *in silico* approach predicted that the MAPK and NF-κB signaling pathways, along with apoptosis-related processes, might serve as central mediators of XNX’s protective effects (Figure 5). This study is subject to several limitations inherent to database-driven network analysis. As highlighted in recent literature, reliance on generic phytochemical databases may introduce “chemical ghosts”—metabolites with high predicted target counts but unconfirmed *in vivo* exposure. In the absence of formulation-specific pharmacokinetic data or multi-omics profiling, our findings should be interpreted as hypothesis-generating rather than definitive mechanistic conclusions, warranting further experimental validation. Nevertheless, in accordance with established guidelines, we have employed several strategies to minimize false-positive results and have conducted a series of experiments to validate our findings. Admittedly, integrating UHPLC-MS/MS analysis of absorbed metabolites with transcriptomic profiling in disease models will constitute a key metabolite of our future work.

**FIGURE 8:**
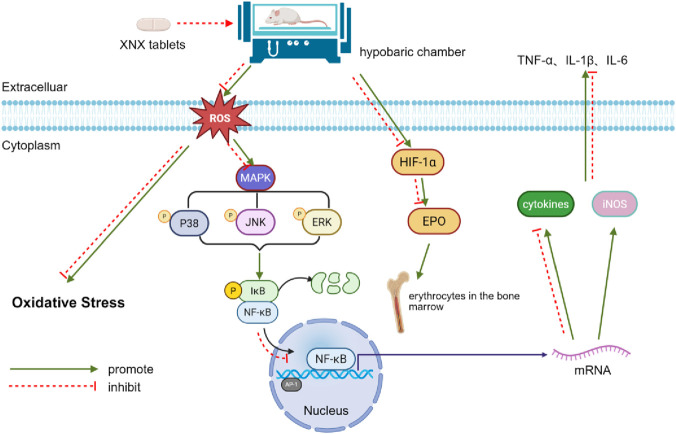
Mechanism of action of XNX tablets.

Building on these predictions, we performed Western blot analyses to experimentally verify the involvement of these candidate pathways. Under HH conditions, complex interactions occur among intracellular oxidative stress (Pena et al., 2022), inflammation, and apoptosis. The MAPK signaling pathway, a canonical oxidative stress-responsive cascade, comprises three major subfamilies, ERK, JNK, and p38 MAPK, which collectively regulate diverse physiological processes, including cell proliferation, apoptosis, inflammation, and immune responses (Osaki and Gama, 2013). Western blot analysis revealed that XNX significantly reduced the ratios of p-MAPK/MAPK, p-JNK/JNK, and p-Akt/Akt (Figures 7B–D), but did not markedly affect p-ERK/ERK (Figure 7E). NF-κB, a downstream effector of MAPK signaling, is normally sequestered in an inactive complex bound to IKBα. Upon phosphorylation of IKBα, NF-κB is released, becomes phosphorylated, translocates to the nucleus, and binds to the promoters of target genes, including various cytokines, chemokines (Lee and Surh, 2012), and prosurvival proteins, thereby regulating immune and inflammatory responses, differentiation, and cell survival (Papa et al., 2006). Our data indicate that XNX confers cardioprotection not only by inhibiting these injury-promoting pathways (NF-κB and p38/JNK MAPK) but also by restoring prosurvival PI3K/AKT signaling, which is suppressed under HH conditions. This coordinated bidirectional modulation, simultaneously suppressing deleterious signals and enhancing protective pathways, likely underlies the comprehensive cardioprotective effects of XNX. During the progression of cardiac hypertrophy induced by pressure overload and hypoxia-related cellular injury, cardiomyocyte apoptosis represents a key pathological mechanism (Kauser et al., 2016; Zhao et al., 2019). Among apoptosis regulators, Bcl-2 is a critical antiapoptotic protein, whereas Bax functions as a proapoptotic factor (Zhu et al., 2010). Apoptotic processes can also amplify ROS production, further exacerbating oxidative stress (Song et al., 2015). Our results demonstrate that XNX treatment downregulates Bax expression, upregulates Bcl-2, and thereby inhibits cardiomyocyte apoptosis. Specifically, HH exposure in mice increased IKBα phosphorylation, activated NF-κB, elevated Bax levels, and suppressed Bcl-2 expression; XNX administration effectively reversed these changes (Figures 7F–I). In summary, XNX mitigates HH-induced oxidative stress, inflammation, and apoptosis in cardiomyocytes through inhibition of the MAPK signaling axis, coupled with restoration of prosurvival PI3K/AKT signaling (Figure 8). These experimentally validated results provide a mechanistic basis for its cardioprotective effects.

In summary, the core innovation of this study lies in demonstrating, through experimental validation, that XNX exerts cardioprotective effects through a dual regulatory mechanism: selectively inhibiting the p38/JNK MAPK-IKBα/NF-κB inflammatory axis while simultaneously activating the PI3K/Akt prosurvival pathway. This coordinated modulation effectively mitigates oxidative stress, inflammation, and apoptosis in cardiomyocytes. These findings provide a novel molecular framework for understanding how TMC formulations can “correct” complex pathological states by orchestrating multiple interconnected signaling networks.

Nevertheless, this study has several limitations. First, although we demonstrated cardiac injury and hematological alterations in response to hypobaric hypoxia, we did not provide additional molecular evidence to establish a definitive causal link between these phenomena. Second, while our network analysis generated useful predictions and our experimental data confirmed the involvement of MAPK signaling, the lack of interventional experiments using specific pathway agonists or antagonists limits definitive validation of the precise molecular targets of XNX. Third, as computational predictions can sometimes yield false positives or be subject to overinterpretation—a concern raised regarding the AKT data—we have been careful to anchor our conclusions primarily on experimental validation. Future studies employing genetic knockdown or pharmacological inhibition approaches will be necessary to establish causality and definitively confirm the molecular mechanisms proposed here.

## 5 Conclusion

Our study demonstrates that XNX protects against HH-induced complications through two primary mechanisms: first, by mitigating cardiac injury via inhibition of the MAPK and NF-κB signaling pathways; and second, by counteracting HAPC through normalization of hematological parameters, suppression of EPO, and promotion of effective erythropoiesis, thereby reducing the generation of bone marrow-derived “ineffective erythrocytes.”

## Data Availability

The original contributions presented in the study are included in the article/Supplementary Material, further inquiries can be directed to the corresponding authors.
