# Correction: Xinnaoxin tablets ameliorate high-altitude polycythemia-associated cardiac injury by regulating the NF-κB, MAPK, and PI3K/AKT signaling pathways

**DOI:** 10.3389/fphar.2026.1900966

**Published:** 2026-06-26

**Authors:** Frontiers Production Office

**Affiliations:** Frontiers Media SA, Lausanne, Switzerland

**Keywords:** Xinnaoxin, hypobaric hypoxia, high-altitude polycythemia, mitogen-activated protein kinase, inflammation, apoptosis

There was a mistake in Figure 5 as published. The incorrect image was used. The corrected Figure 5 appears below.

**FIGURE 5:**
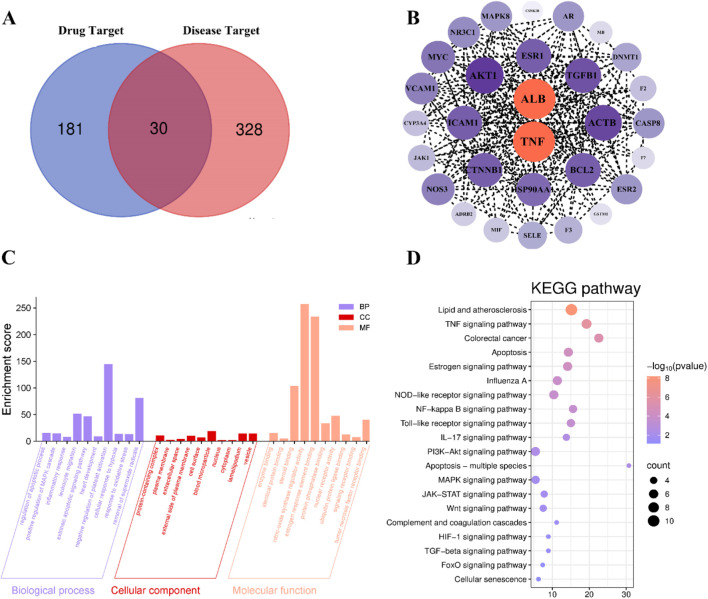
An integrative network analysis approach to elucidate the therapeutic mechanisms of XNX against HAPC. **(A)** Venn diagram of the shared targets between XNX and HAPC. **(B)** PPI network of the common targets. Node size and color intensity represent the degree of connectivity. **(C)** GO functional enrichment analysis. **(D)** KEGG pathway enrichment analysis.

The original article has been updated.

