# Supplementary material for: Xinnaoxin tablets ameliorate high-altitude polycythemia-associated cardiac injury by regulating the NF-κB, MAPK, and PI3K/AKT signaling pathways
Source: Front Pharmacol. 2026 May 28;17:1754806. doi: 10.3389/fphar.2026.1754806 (PMC13253415; doi:10.3389/fphar.2026.1754806)
Supplement: Supplementary file 1 [file DataSheet7.pdf]

botanical drug	components	potential targets
Lycii Fructus	Sitosterol alpha1	PTGS2;PGR;NR3C2
Lycii Fructus	Mandenol	PTGS1;NCOA2
Lycii Fructus	Stigmasterol	CHRM3; CHRM1; ADRB1; SCN5A; ADRA2A; RXRA; HTR2A; SLC6A2; ADRA1D; CHRM2; ADRA1B; SLC6A3; ADRB2; AKR1B1; PLAU; LTA4H; MAOB; MAOA; CHRNA7; PKIA; CTRB1; NCOA1;
Lycii Fructus	beta-sitosterol	CHRM3; ADRB1; SCN5A; ADRA2A; HTR2A; ADRA1D; SLC6A3; ADRB2; PLAU; DRD1; KCNH2; BCL2; CHRM4; PDE3A; CHRNA2; SLC6A4; OPRM1; PON1; JUN; HSP90AA1; MAP2; BAX; CASP3; CASP8; CASP9; PRKCA; TGFB1
Lycii Fructus	atropine	CHRM5; HTR1A; ADRA2C; OPRD1; HRH1; HTR2C; ADRA2B; DRD2; HTR1B; DRD5
Lycii Fructus	glycitein	NOS2; ESR1; AR; PPARG; ESR2; MMP13; MAPK14; GSK3B; MMP8; CDK2; CHEK1; APP; PRSS1; CCNA2; CALM3
Lycii Fructus	7-Dehydrocholesterol	NR3C1
Lycii Fructus, Hippophae Fructus	sitosterol	F2; ESR1; NOS3; F7; PTPN1; ESR2; DPP4; PYGM; PPARD; MAPK14; GSK3B; CDK2; MAOB; XDH; CHEK1; PRSS1; CCNA2; GRIA2; NCOA1; CALM3; NCF1; OLR1;
Hippophae Fructus	Beta-carotene	DRD1; KCNH2; BCL2; CHRM4; PDE3A; CHRNA2; SLC6A4; OPRM1; PON1; JUN; HSP90AA1; MAP2; BAX; CASP3; CASP8; CASP9; PRKCA; TGFB1
Hippophae Fructus, Rhodiolae Crenulatae Radix et Rhizoma	kaempferol	INSR; CHRM1; ALOX5; SLC6A2; CHRM2; ADRA1B; AKR1C3; TNF; TOP2A; SELE; CDK1; VCAM1; MAPK8; GSTM1; AHR; GSTM2; PSMD3; AHSA1; SLPI; BAX; CYP1A1; CYP1B1; HAS2; IKBKB; ICAM1; NR1I2; NR1I3; STAT1; SLC2A4; DIO1; AHR; AKR1C1; AKT1; ATP5A1; ATP5B; ATP5C1; CA1; CA12; CA14; CA2; CA3; CA4; CA5A; CA5B; CA6; CA7; CA9; CBR1; CDK6; CEBPB; COMT; CSNK2A1; CSNK2B; CYP19A1; CYP1B1; DHFRL1; DNMT1; EIF3F; ESR1; ESR2; GPER1; HCK; HIBCH; HSP90AA1; HSPA2; JAK1; KANSL3; MTTP; NCOA1; NCOA2; NQO2; NR1I2; PIK3CG; PIM1; PRKACA; PRKCA; PRKCB; PTK2B; RUVBL2; SF3B3; SHBG; SOAT1; SOAT2; SQLE; STK17B; SYK; TOP2A; UBA1; UGT3A1; ESRRB; GABRA1; ESRRA; GABRA2; GABRA3 ; GABRA4; GABRA5; GABRA6; GABRG1; GABRG2; GABRG3;
Hippophae Fructus	(+)-catechin	LACTBL1
Rhodiolae Crenulatae Radix et	7-Hydroxycoumarin	IGHG1; SHBG; CYP2A6;
Rhodiolae Crenulatae Radix et	Caffeic Acid	LCN2; MIF; CTRB1

Rhodiolae Crenulatae Radix et	Gallic Acid	ATP5A1; ATP5B; ATP5C1; LCN2; MIF; PTGS2; PTGS1; AMY2A; AMY2B;
Rhodiolae Crenulatae Radix et Rhizoma	Rhodioloside	AR; BAMF_RS28815; CYP2B6; GCK; GLT6D1; GLTP; GNPDA1; HK1; IFNB1; KRTAP5-2; KRTAP5-3; LCTL; LGALS2; LGALS3; LGALS7; MB; NCAN; NUDT9; PTGS1; PYGL; PYGM; SFTPD; SIGLEC1; SMARCA5; TM0024 TYR; ABO; AMY1A;
