# Supplementary material for: Xinnaoxin tablets ameliorate high-altitude polycythemia-associated cardiac injury by regulating the NF-κB, MAPK, and PI3K/AKT signaling pathways
Source: Front Pharmacol. 2026 May 28;17:1754806. doi: 10.3389/fphar.2026.1754806 (PMC13253415; doi:10.3389/fphar.2026.1754806)
Supplement: Supplementary file 2 [file DataSheet2.pdf]

Botanical drugs		Botanical drugs		Botanical drugs	
gouqizi		shaji		hongjingtian	
Molecule Name	Target Name	Molecule Name	Target Name	Molecule Name	Target Name
Sitosterol alpha	PTGS2	pelargonidin	NOS2	7-Hydroxycoum	IGHG1
Sitosterol alpha	PGR	pelargonidin	PTGS1	7-Hydroxycoum	SHBG
Sitosterol alpha	NR3C2	pelargonidin	AR	7-Hydroxycoum	CYP2A6
Mandenol	PTGS1	pelargonidin	PPARG	Caffeic Acid	LCN2
Mandenol	NCOA2	pelargonidin	PTGS2	Caffeic Acid	MIF
Stigmasterol	CHRM3	pelargonidin	CA2	Caffeic Acid	CTRB1
Stigmasterol	CHRM1	pelargonidin	RXRA	Kaempferol	AHR
Stigmasterol	ADRB1	pelargonidin	ACHE	Kaempferol	AKR1C1
Stigmasterol	SCN5A	pelargonidin	PGR	Kaempferol	AKT1
Stigmasterol	ADRA2A	pelargonidin	NR3C2	Kaempferol	ATP5A1
Stigmasterol	RXRA	pelargonidin	NR3C1	Kaempferol	ATP5B
Stigmasterol	HTR2A	pelargonidin	HSP90AA1	Kaempferol	ATP5C1
Stigmasterol	SLC6A2	pelargonidin	PKIA	Kaempferol	CA1
Stigmasterol	ADRA1D	pelargonidin	NCOA2	Kaempferol	CA12
Stigmasterol	CHRM2	rhein	AKR1B1	Kaempferol	CA14
Stigmasterol	ADRA1B	rhein	JUN	Kaempferol	CA2
Stigmasterol	SLC6A3	beta-carotene	BCL2	Kaempferol	CA3
Stigmasterol	ADRB2	beta-carotene	MMP2	Kaempferol	CA4
Stigmasterol	AKR1B1	beta-carotene	MMP1	Kaempferol	CA5A
Stigmasterol	PLAU	beta-carotene	GJA1	Kaempferol	CA5A
Stigmasterol	LTA4H	beta-carotene	F3	Kaempferol	CA5B
Stigmasterol	MAOB	beta-carotene	CYP3A4	Kaempferol	CA6
Stigmasterol	MAOA	beta-carotene	HMOX1	Kaempferol	CA7
Stigmasterol	CHRNA7	beta-carotene	CASP7	Kaempferol	CA9
Stigmasterol	PKIA	beta-carotene	CASP3	Kaempferol	CBR1
Stigmasterol	CTRB1	beta-carotene	CASP8	Kaempferol	CDK6
Stigmasterol	NCOA1	beta-carotene	CASP9	Kaempferol	CEBPB
beta-sitosterol	DRD1	beta-carotene	CTNNB1	Kaempferol	COMT
beta-sitosterol	KCNH2	beta-carotene	CAV1	Kaempferol	CSNK2A1
beta-sitosterol	BCL2	beta-carotene	MYC	Kaempferol	CSNK2B
beta-sitosterol	CHRM4	beta-carotene	AKT1	Kaempferol	CYP19A1
beta-sitosterol	PDE3A	beta-carotene	ALB	Kaempferol	CYP1B1
beta-sitosterol	CHRNA2	sitosterol	F2	Kaempferol	DHFRL1
beta-sitosterol	SLC6A4	sitosterol	ESR1	Kaempferol	DNMT1
beta-sitosterol	OPRM1	sitosterol	NOS3	Kaempferol	EIF3F
beta-sitosterol	PON1	sitosterol	F7	Kaempferol	ESR1
beta-sitosterol	JUN	sitosterol	PTPN1	Kaempferol	ESR2
beta-sitosterol	HSP90AA1	sitosterol	ESR2	Kaempferol	GPER1
beta-sitosterol	MAP2	sitosterol	DPP4	Kaempferol	HCK
beta-sitosterol	BAX	sitosterol	PYGM	Kaempferol	HIBCH
beta-sitosterol	CASP3	sitosterol	PPARD	Kaempferol	HSP90AA1
beta-sitosterol	CASP8	sitosterol	MAPK14	Kaempferol	HSPA2
beta-sitosterol	CASP9	sitosterol	GSK3B	Kaempferol	JAK1
beta-sitosterol	PRKCA	sitosterol	CDK2	Kaempferol	KANSL3
beta-sitosterol	TGFB1	sitosterol	MAOB	Kaempferol	MTTP
atropine	CHRM5	sitosterol	XDH	Kaempferol	NCOA1
atropine	HTR1A	sitosterol	CHEK1	Kaempferol	NCOA2
atropine	ADRA2C	sitosterol	PRSS1	Kaempferol	NQO2
atropine	OPRD1	sitosterol	CCNA2	Kaempferol	NR1I2
atropine	HRH1	sitosterol	GRIA2	Kaempferol	PIK3CG

atropine	HTR2C	sitosterol	NCOA1	Kaempferol	PIM1
atropine	ADRA2B	sitosterol	CALM3	Kaempferol	PRKACA
atropine	DRD2	sitosterol	NCF1	Kaempferol	PRKCA
atropine	HTR1B	sitosterol	OLR1	Kaempferol	PRKCB
glycitein	NOS2	sitosterol	RELA	Kaempferol	PTK2B
glycitein	ESR1	kaempferol	INSR	Kaempferol	RUVBL2
glycitein	AR	kaempferol	CHRM1	Kaempferol	SF3B3
glycitein	PPARG	kaempferol	ALOX5	Kaempferol	SHBG
glycitein	ESR2	kaempferol	SLC6A2	Kaempferol	SOAT1
glycitein	MMP13	kaempferol	CHRM2	Kaempferol	SOAT2
glycitein	MAPK14	kaempferol	ADRA1B	Kaempferol	SQLE
glycitein	GSK3B	kaempferol	AKR1C3	Kaempferol	STK17B
glycitein	MMP8	kaempferol	TNF	Kaempferol	SYK
glycitein	CDK2	kaempferol	TOP2A	Kaempferol	TOP2A
glycitein	CHEK1	kaempferol	SELE	Kaempferol	UBA1
glycitein	APP	kaempferol	CDK1	Kaempferol	UGT3A1
glycitein	PRSS1	kaempferol	VCAM1	Kaempferol	ESRRB
glycitein	CCNA2	kaempferol	MAPK8	Kaempferol	GABRA1
glycitein	CALM3	kaempferol	GSTM1	Kaempferol	ESRRA
6-Fluoroindole-	NR3C1	kaempferol	AHR	Kaempferol	GABRA2
7-O-Methyllute	TOP2A	kaempferol	GSTM2	Kaempferol	GABRA3
Atropine	DRD5	kaempferol	PSMD3	Kaempferol	GABRA4
quercetin	INSR	kaempferol	AHSA1	Kaempferol	GABRA5
quercetin	F2	kaempferol	SLPI	Kaempferol	GABRA6
quercetin	F10	kaempferol	BAX	Kaempferol	GABRG1
quercetin	ALOX5	kaempferol	CYP1A1	Kaempferol	GABRG2
quercetin	NOS3	kaempferol	CYP1B1	Kaempferol	GABRG3
quercetin	F7	kaempferol	HAS2	Kaempferol	ACTB
quercetin	ODC1	kaempferol	IKBKB	Kaempferol	AKR1C1
quercetin	ACHE	kaempferol	ICAM1	Gallic Acid	ATP5A1
quercetin	ACACA	kaempferol	NR1I2	Gallic Acid	ATP5B
quercetin	MMP2	kaempferol	NR1I3	Gallic Acid	ATP5C1
quercetin	TNF	kaempferol	STAT1	Gallic Acid	LCN2
quercetin	EGFR	kaempferol	SLC2A4	Gallic Acid	MIF
quercetin	MGAM	kaempferol	DIO1	Gallic Acid	PTGS2
quercetin	DPP4	Stigmasterol	CHRM3	Gallic Acid	PTGS1
quercetin	IL6	Stigmasterol	ADRB1	Gallic Acid	AMY2A
quercetin	MMP1	Stigmasterol	SCN5A	Gallic Acid	AMY2B
quercetin	MAPK1	Stigmasterol	ADRA2A	Rhodioloside	AR
quercetin	CTSD	Stigmasterol	HTR2A	Rhodioloside	BAMF_RS2881
quercetin	IFNG	Stigmasterol	ADRA1D	Rhodioloside	CYP2B6
quercetin	IL1B	Stigmasterol	SLC6A3	Rhodioloside	GCK
quercetin	SELE	Stigmasterol	ADRB2	Rhodioloside	GLT6D1
quercetin	MPO	Stigmasterol	PLAU	Rhodioloside	GLTP
quercetin	CDK1	Stigmasterol	LTA4H	Rhodioloside	GNPDA1
quercetin	PLAT	Stigmasterol	MAOA	Rhodioloside	HK1
quercetin	GJA1	(+)-catechin	LACTBL1	Rhodioloside	IFNB1
quercetin	VCAM1	quercetin	KCNH2	Rhodioloside	KRTAP5-2
quercetin	MMP3	quercetin	F10	Rhodioloside	KRTAP5-3
quercetin	THBD	quercetin	ODC1	Rhodioloside	LCTL
quercetin	F3	quercetin	ACACA	Rhodioloside	LGALS2
quercetin	NQO1	quercetin	EGFR	Rhodioloside	LGALS3
quercetin	XDH	quercetin	MGAM	Rhodioloside	LGALS7

quercetin	PTGER3	quercetin	IL6	Rhodioloside	MB
quercetin	SOD1	quercetin	MAPK1	Rhodioloside	NCAN
quercetin	CYP3A4	quercetin	PON1	Rhodioloside	NUDT9
quercetin	TP53	quercetin	CTSD	Rhodioloside	PTGS1
quercetin	TOP1	quercetin	IFNG	Rhodioloside	PYGL
quercetin	HMOX1	quercetin	IL1B	Rhodioloside	PYGM
quercetin	COL3A1	quercetin	MPO	Rhodioloside	SFTPD
quercetin	RB1	quercetin	PLAT	Rhodioloside	SIGLEC1
quercetin	GSTM1	quercetin	MMP3	Rhodioloside	SMARCA5
quercetin	HSPA5	quercetin	THBD	Rhodioloside	TM0024 TYR
quercetin	ACPP	quercetin	NQO1	Rhodioloside	ABO
quercetin	AHR	quercetin	PTGER3	Rhodioloside	AMY1A
quercetin	GSTM2	quercetin	SOD1		
quercetin	IL2	quercetin	TP53		
quercetin	PSMD3	quercetin	TOP1		
quercetin	AHSA1	quercetin	COL3A1		
quercetin	ABCG2	quercetin	RB1		
quercetin	BIRC5	quercetin	HSPA5		
quercetin	BCL2L1	quercetin	ACPP		
quercetin	CAV1	quercetin	IL2		
quercetin	CD40LG	quercetin	ABCG2		
quercetin	CLDN4	quercetin	BIRC5		
quercetin	CRP	quercetin	BCL2L1		
quercetin	CXCL10	quercetin	CD40LG		
quercetin	CXCL11	quercetin	CLDN4		
quercetin	CXCL2	quercetin	CRP		
quercetin	CDKN1A	quercetin	CXCL10		
quercetin	CYP1A1	quercetin	CXCL11		
quercetin	CYP1B1	quercetin	CXCL2		
quercetin	DCAF5	quercetin	CDKN1A		
quercetin	DUOX2	quercetin	DCAF5		
quercetin	ELK1	quercetin	DUOX2		
quercetin	EIF6	quercetin	ELK1		
quercetin	CCND1	quercetin	EIF6		
quercetin	CCNB1	quercetin	CCND1		
quercetin	HSF1	quercetin	CCNB1		
quercetin	HSPB1	quercetin	HSF1		
quercetin	HK2	quercetin	HSPB1		
quercetin	NKX3-1	quercetin	HK2		
quercetin	HAS2	quercetin	NKX3-1		
quercetin	HIF1A	quercetin	HIF1A		
quercetin	CHUK	quercetin	CHUK		
quercetin	IGFBP3	quercetin	IGFBP3		
quercetin	IGF2	quercetin	IGF2		
quercetin	ICAM1	quercetin	IRF1		
quercetin	IRF1	quercetin	IL10		
quercetin	IL10	quercetin	IL1A		
quercetin	IL1A	quercetin	CXCL8		
quercetin	CXCL8	quercetin	MMP9		
quercetin	MMP9	quercetin	NFKBIA		
quercetin	MYC	quercetin	NFE2L2		
quercetin	NCF1	quercetin	SPP1		
quercetin	NFKBIA	quercetin	PPARA		

quercetin	NFE2L2	quercetin	SERPINE1		
quercetin	NR1I2	quercetin	PARP1		
quercetin	NR1I3	quercetin	PCOLCE		
quercetin	SPP1	quercetin	RUNX1T1		
quercetin	PPARA	quercetin	PRKCA		
quercetin	PPARD	quercetin	PRKCB		
quercetin	SERPINE1	quercetin	FOS		
quercetin	PARP1	quercetin	NPEPPS		
quercetin	PCOLCE	quercetin	RASSF1		
quercetin	RUNX1T1	quercetin	RASA1		
quercetin	PRKCB	quercetin	ERBB2		
quercetin	FOS	quercetin	ERBB3		
quercetin	NPEPPS	quercetin	RUNX2		
quercetin	AKT1	quercetin	CHEK2		
quercetin	RASSF1	quercetin	E2F1		
quercetin	RASA1	quercetin	E2F2		
quercetin	ERBB2	quercetin	TGFB1		
quercetin	ERBB3				
quercetin	RUNX2				
quercetin	CHEK2				
quercetin	STAT1				
quercetin	SLC2A4				
quercetin	E2F1				
quercetin	E2F2				
quercetin	RELA				
quercetin	DIO1				

