# Supplementary material for: Xinnaoxin tablets ameliorate high-altitude polycythemia-associated cardiac injury by regulating the NF-κB, MAPK, and PI3K/AKT signaling pathways
Source: Front Pharmacol. 2026 May 28;17:1754806. doi: 10.3389/fphar.2026.1754806 (PMC13253415; doi:10.3389/fphar.2026.1754806)
Supplement: Supplementary file 3 [file DataSheet4.pdf]

Molecule	Name	Canonical SMILES	Formula	MW	#Heavy atoms	#Aromatic heavy atoms	Fraction Csp3	#Rotatable bonds	#H-bond acceptors	#H-bond donors	MR	TPSA	iLOGP	XLOGP3	WLOGP	MLOGP	Silicos-IT Log P	Consensus Log P
Molecule 1	Sitosterol alpha1	C/C=C(\C(C)C)/CC[C@H]([C@H]1CC[C@@H]2[C@]1(C)CC[C@H]1C2=CC[C@@H]2[C@]1(C)CC[C@@H]([C@H]2C)O)C	C30H50O	426.72	31	0	0.87	5	1	1	137.56	20.23	5.24	9.03	8.19	6.82	7	7.26
Molecule 2	Mandenol	CCCCC/C=C\C/C=C\CCCCCCCC(=O)OCC	C20H36O2	308.5	22	0	0.75	16	2	0	98.59	26.3	5.24	9.03	6.36	6.82	7	7.26
Molecule 3	Stigmasterol	CC[C@@H](C(C)C)/C=C/[C@H]([C@H]1CC[C@@H]2[C@]1(C)CC[C@H]1[C@H]2CC=C2[C@]1(C)CC[C@@H](C2)O)C	C29H48O	412.69	30	0	0.86	5	1	1	132.75	20.23	5.08	8.56	7.8	6.62	6.86	6.98
Molecule 4	beta-sitosterol	CC[C@@H](C(C)C)CC[C@H]([C@H]1CC[C@@H]2[C@]1(C)CC[C@H]1[C@H]2CC=C2[C@]1(C)CC[C@@H](C2)O)C	C29H50O	414.71	30	0	0.93	6	1	1	133.23	20.23	5.05	9.34	8.02	6.73	7.04	7.24
Molecule 5	atropine	OCC(c1ccccc1)C(=O)O[C@@H]1C[C@@H]2CC[C@H](C1)N2C	C17H23NO3	289.37	21	6	0.59	5	4	1	84.51	49.77	2.61	1.83	1.55	2.02	2.03	2.01
Molecule 6	glycitein	COc1cc2c(cc1O)occ(c2=O)c1ccc(cc1)O	C16H12O5	284.26	21	16	0.06	2	5	2	78.46	79.9	2.36	2.44	2.88	0.77	3.03	2.3
Molecule 7	7-Dehydrocholesterol	CC(CCC[C@H]([C@H]1CC[C@@H]2[C@]1(C)CC[C@H]1C2=CC=C2[C@]1(C)CC[C@@H](C2)O)C)C	C27H44O	384.64	28	0	0.85	5	1	1	123.14	20.23	4.9	7.96	7.31	6.23	6.39	6.56
Molecule 8	7-O-Methylluteolin-6-C-beta-glucoside_qt	COc1cc(O)c2c(c1)oc(cc2=O)c1ccc(c(c1)O)O	C16H12O6	300.26	22	16	0.06	2	6	3	80.48	100.13	2.45	2.86	2.59	0.22	2.55	2.13
Molecule 9	quercetin	Oc1cc(O)c2c(c1)oc(c(c2=O)O)c1ccc(c(c1)O)O	C15H10O7	302.24	22	16	0	1	7	5	78.03	131.36	1.63	1.54	1.99	-0.56	1.54	1.23
Molecule 10	sitosterol	CC[C@@H](C(C)C)CC[C@H]([C@H]1CC[C@@H]2[C@]1(C)CC[C@H]1[C@H]2CC=C2[C@]1(C)CC[C@@H](C2)O)C	C29H50O	414.71	30	0	0.93	6	1	1	133.23	20.23	5.05	9.34	8.02	6.73	7.04	7.24
Molecule 11	pelargonidin	Oc1ccc(cc1)c1[o+]c2cc(O)cc(c2cc1O)O	C15H11O5+	271.24	20	16	0	1	5	4	74.15	94.06	-2.44	2.29	3.2	0.86	0.73	0.93
Molecule 12	Beta-carotene	C/C(=C\C=C\C=C(\C=C\C=C(\C=C\C1=C(C)CCCC1(C)C)/C)/C)/C=C/C=C(/C=C/C1=C(C)CCCC1(C)C)\C	C40H56	536.87	40	0	0.45	10	0	0	184.43	0	-2.44	2.29	12.61	0.86	0.73	0.93
Molecule 13	kaempferol	Oc1ccc(cc1)c1oc2cc(O)cc(c2c(=O)c1O)O	C15H10O6	286.24	21	16	0	1	6	4	76.01	111.13	1.7	1.9	2.28	-0.03	2.03	1.58
Molecule 14	(+)-catechin	Oc1cc2O[C@H](c3ccc(c(c3)O)O)[C@H](Cc2c(c1)O)O	C15H14O6	290.27	21	12	0.2	1	6	5	74.33	110.38	1.33	0.36	1.22	0.24	0.98	0.83
Molecule 15	7-Hydroxycoumarin	Oc1ccc2c(c1)oc(=O)cc2	C9H6O3	162.14	12	10	0	0	3	1	44.51	50.44	1.44	1.58	1.5	1.04	1.97	1.51
Molecule 16	Caffeic Acid	OC(=O)/C=C/c1ccc(c(c1)O)O	C9H8O4	180.16	13	6	0	2	4	3	47.16	77.76	0.97	1.15	1.09	0.7	0.75	0.93
Molecule 17	Gallic Acid	OC(=O)c1cc(O)c(c(c1)O)O	C7H6O5	170.12	12	6	0	1	5	4	39.47	97.99	0.21	0.7	0.5	-0.16	-0.2	0.21
Molecule 18	Rhodioloside	OC[C@H]1O[C@@H](OCCc2ccc(cc2)O)[C@@H]([C@H]([C@@H]1O)O)O	C14H20O7	300.3	21	6	0.57	5	7	5	71.78	119.61	1.04	-1.05	-1.25	-1.22	-0.48	-0.59

Molecule	Name	Canonical SMILES	Formula	ESOL Log S	ESOL Solubility (mg/ml)	ESOL Solubility (mol/l)	ESOL Class	Ali Log S	Ali Solubility (mg/ml)	Ali Solubility (mol/l)	Ali Class	Silicos-IT LogSw	Silicos-IT Solubility (mg/ml)	Silicos-IT Solubility (mol/l)	Silicos-IT class	GI absorption	BBB permeant	Pgp substrate
Molecule 1	Sitosterol alpha1	C/C=C(\C(C)C)/CC[C@H]([C@H]1CC[C@@H]2[C@]1(C)CC[C@H]1C2=CC[C@@H]2[C@]1(C)CC[C@@H]([C@H]2C)O)C	C30H50O	-7.84	6.10E-06	1.43E-08	Poorly sol	-9.35	1.92E-07	4.50E-10	Poorly sol	-5.97	4.57E-04	1.07E-06	Moderate	Low	No	No
Molecule 2	Mandenol	CCCCC/C=C\C/C=C\CCCCCCCC(=O)OCC	C20H36O2	-7.84	6.10E-06	1.43E-08	Poorly sol	-9.35	1.92E-07	4.50E-10	Poorly sol	-5.97	4.57E-04	1.07E-06	Moderate	Low	No	No
Molecule 3	Stigmasterol	CC[C@@H](C(C)C)/C=C/[C@H]([C@H]1CC[C@@H]2[C@]1(C)CC[C@H]1[C@H]2CC=C2[C@]1(C)CC[C@@H](C2)O)C	C29H48O	-7.46	1.43E-05	3.46E-08	Poorly sol	-8.86	5.71E-07	1.38E-09	Poorly sol	-5.47	1.40E-03	3.39E-06	Moderate	Low	No	No
Molecule 4	beta-sitosterol	CC[C@@H](C(C)C)CC[C@H]([C@H]1CC[C@@H]2[C@]1(C)CC[C@H]1[C@H]2CC=C2[C@]1(C)CC[C@@H](C2)O)C	C29H50O	-7.9	5.23E-06	1.26E-08	Poorly sol	-9.67	8.90E-08	2.15E-10	Poorly sol	-6.19	2.69E-04	6.49E-07	Poorly sol	Low	No	No
Molecule 5	atropine	OCC(c1ccccc1)C(=O)O[C@@H]1C[C@@H]2CC[C@H](C1)N2C	C17H23NO3	-2.67	6.21E-01	2.15E-03	Soluble	-2.5	9.25E-01	3.20E-03	Soluble	-2.93	3.41E-01	1.18E-03	Soluble	High	Yes	No
Molecule 6	glycitein	COc1cc2c(cc1O)occ(c2=O)c1ccc(cc1)O	C16H12O5	-3.57	7.63E-02	2.68E-04	Soluble	-3.76	4.93E-02	1.73E-04	Soluble	-5.1	2.25E-03	7.91E-06	Moderate	High	No	No
Molecule 7	7-Dehydrocholesterol	CC(CCC[C@H]([C@H]1CC[C@@H]2[C@]1(C)CC[C@H]1C2=CC=C2[C@]1(C)CC[C@@H](C2)O)C)C	C27H44O	-6.91	4.74E-05	1.23E-07	Poorly sol	-8.24	2.23E-06	5.81E-09	Poorly sol	-5.76	6.70E-04	1.74E-06	Moderate	Low	No	No
Molecule 8	7-O-Methylluteolin-6-C-beta-glucoside_qt	COc1cc(O)c2c(c1)oc(cc2=O)c1ccc(c(c1)O)O	C16H12O6	-3.91	3.70E-02	1.23E-04	Soluble	-4.62	7.17E-03	2.39E-05	Moderate	-4.52	9.07E-03	3.02E-05	Moderate	High	No	No
Molecule 9	quercetin	Oc1cc(O)c2c(c1)oc(c(c2=O)O)c1ccc(c(c1)O)O	C15H10O7	-3.16	2.11E-01	6.98E-04	Soluble	-3.91	3.74E-02	1.24E-04	Soluble	-3.24	1.73E-01	5.73E-04	Soluble	High	No	No
Molecule 10	sitosterol	CC[C@@H](C(C)C)CC[C@H]([C@H]1CC[C@@H]2[C@]1(C)CC[C@H]1[C@H]2CC=C2[C@]1(C)CC[C@@H](C2)O)C	C29H50O	-7.9	5.23E-06	1.26E-08	Poorly sol	-9.67	8.90E-08	2.15E-10	Poorly sol	-6.19	2.69E-04	6.49E-07	Poorly sol	Low	No	No
Molecule 11	pelargonidin	Oc1ccc(cc1)c1[o+]c2cc(O)cc(c2cc1O)O	C15H11O5+	-3.49	8.77E-02	3.23E-04	Soluble	-3.9	3.39E-02	1.25E-04	Soluble	-3.24	1.58E-01	5.81E-04	Soluble	High	No	Yes
Molecule 12	Beta-carotene	C/C(=C\C=C\C=C(\C=C\C=C(\C=C\C1=C(C)CCCC1(C)C)/C)/C)/C=C/C=C(/C=C/C1=C(C)CCCC1(C)C)\C	C40H56	-3.49	8.77E-02	3.23E-04	Soluble	-3.9	3.39E-02	1.25E-04	Soluble	-3.24	1.58E-01	5.81E-04	Soluble	High	No	Yes
Molecule 13	kaempferol	Oc1ccc(cc1)c1oc2cc(O)cc(c2c(=O)c1O)O	C15H10O6	-3.31	1.40E-01	4.90E-04	Soluble	-3.86	3.98E-02	1.39E-04	Soluble	-3.82	4.29E-02	1.50E-04	Soluble	High	No	No
Molecule 14	(+)-catechin	Oc1cc2O[C@H](c3ccc(c(c3)O)O)[C@H](Cc2c(c1)O)O	C15H14O6	-2.22	1.74E+00	5.98E-03	Soluble	-2.24	1.66E+00	5.72E-03	Soluble	-2.14	2.09E+00	7.19E-03	Soluble	High	No	Yes
Molecule 15	7-Hydroxycoumarin	Oc1ccc2c(c1)oc(=O)cc2	C9H6O3	-2.46	5.66E-01	3.49E-03	Soluble	-2.25	9.12E-01	5.62E-03	Soluble	-3.03	1.53E-01	9.42E-04	Soluble	High	Yes	No
Molecule 16	Caffeic Acid	OC(=O)/C=C/c1ccc(c(c1)O)O	C9H8O4	-1.89	2.32E+00	1.29E-02	Very solub	-2.38	7.55E-01	4.19E-03	Soluble	-0.71	3.51E+01	1.95E-01	Soluble	High	No	No
Molecule 17	Gallic Acid	OC(=O)c1cc(O)c(c(c1)O)O	C7H6O5	-1.64	3.90E+00	2.29E-02	Very solub	-2.34	7.86E-01	4.62E-03	Soluble	-0.04	1.55E+02	9.10E-01	Soluble	High	No	No
Molecule 18	Rhodioloside	OC[C@H]1O[C@@H](OCCc2ccc(cc2)O)[C@@H]([C@H]([C@@H]1O)O)O	C14H20O7	-0.92	3.60E+01	1.20E-01	Very solub	-0.97	3.19E+01	1.06E-01	Very solub	-0.44	1.08E+02	3.61E-01	Soluble	High	No	No

Molecule	Name	Canonical SMILES	Formula	CYP1A2 inhibitor	CYP2C19 inhibitor	CYP2C9 inhibitor	CYP2D6 inhibitor	CYP3A4 inhibitor	log Kp (cm/s)	Lipinski #violations	Ghose #violations	Veber #violations	Egan #violations	Muegge #violations	Bioavailability Score	PAINS #alerts	Brenk #alerts	Leadlikeness #violations	Synthetic Accessibility
Molecule 1	Sitosterol alpha1	C/C=C(\C(C)C)/CC[C@H]([C@H]1CC[C@@H]2[C@]1(C)CC[C@H]1C2=CC[C@@H]2[C@]1(C)CC[C@@H]([C@H]2C)O)C	C30H50O	No	No	No	No	No	-2.49	1	3	0	1	2	0.55	0	1	2	6.22
Molecule 2	Mandenol	CCCCC/C=C\C/C=C\CCCCCCCC(=O)OCC	C20H36O2	No	No	No	No	No	-2.49	1	3	0	1	2	0.55	0	1	2	6.22
Molecule 3	Stigmasterol	CC[C@@H](C(C)C)/C=C/[C@H]([C@H]1CC[C@@H]2[C@]1(C)CC[C@H]1[C@H]2CC=C2[C@]1(C)CC[C@@H](C2)O)C	C29H48O	No	No	Yes	No	No	-2.74	1	3	0	1	2	0.55	0	1	2	6.21
Molecule 4	beta-sitosterol	CC[C@@H](C(C)C)CC[C@H]([C@H]1CC[C@@H]2[C@]1(C)CC[C@H]1[C@H]2CC=C2[C@]1(C)CC[C@@H](C2)O)C	C29H50O	No	No	No	No	No	-2.2	1	3	0	1	2	0.55	0	1	2	6.3
Molecule 5	atropine	OCC(c1ccccc1)C(=O)O[C@@H]1C[C@@H]2CC[C@H](C1)N2C	C17H23NO3	No	No	No	Yes	No	-6.77	0	0	0	0	0	0.55	0	0	0	4.33
Molecule 6	glycitein	COc1cc2c(cc1O)occ(c2=O)c1ccc(cc1)O	C16H12O5	Yes	No	No	Yes	Yes	-6.3	0	0	0	0	0	0.55	0	0	0	2.95
Molecule 7	7-Dehydrocholesterol	CC(CCC[C@H]([C@H]1CC[C@@H]2[C@]1(C)CC[C@H]1C2=CC=C2[C@]1(C)CC[C@@H](C2)O)C)C	C27H44O	No	No	Yes	No	No	-2.99	1	2	0	1	2	0.55	0	0	2	6.45
Molecule 8	7-O-Methylluteolin-6-C-beta-glucoside_qt	COc1cc(O)c2c(c1)oc(cc2=O)c1ccc(c(c1)O)O	C16H12O6	Yes	No	Yes	Yes	Yes	-6.1	0	0	0	0	0	0.55	1	1	0	3.1
Molecule 9	quercetin	Oc1cc(O)c2c(c1)oc(c(c2=O)O)c1ccc(c(c1)O)O	C15H10O7	Yes	No	No	Yes	Yes	-7.05	0	0	0	0	0	0.55	1	1	0	3.23
Molecule 10	sitosterol	CC[C@@H](C(C)C)CC[C@H]([C@H]1CC[C@@H]2[C@]1(C)CC[C@H]1[C@H]2CC=C2[C@]1(C)CC[C@@H](C2)O)C	C29H50O	No	No	No	No	No	-2.2	1	3	0	1	2	0.55	0	1	2	6.3
Molecule 11	pelargonidin	Oc1ccc(cc1)c1[o+]c2cc(O)cc(c2cc1O)O	C15H11O5+	Yes	No	No	Yes	No	-6.33	0	0	0	0	0	0.55	0	1	0	3.04
Molecule 12	Beta-carotene	C/C(=C\C=C\C=C(\C=C\C=C(\C=C\C1=C(C)CCCC1(C)C)/C)/C)/C=C/C=C(/C=C/C1=C(C)CCCC1(C)C)\C	C40H56	Yes	No	No	Yes	No	-6.33	0	0	0	0	0	0.55	0	1	0	3.04
Molecule 13	kaempferol	Oc1ccc(cc1)c1oc2cc(O)cc(c2c(=O)c1O)O	C15H10O6	Yes	No	No	Yes	Yes	-6.7	0	0	0	0	0	0.55	0	0	0	3.14
Molecule 14	(+)-catechin	Oc1cc2O[C@H](c3ccc(c(c3)O)O)[C@H](Cc2c(c1)O)O	C15H14O6	No	No	No	No	No	-7.82	0	0	0	0	0	0.55	1	1	0	3.5
Molecule 15	7-Hydroxycoumarin	Oc1ccc2c(c1)oc(=O)cc2	C9H6O3	Yes	No	No	No	No	-6.17	0	1	0	0	1	0.55	0	1	1	2.56
Molecule 16	Caffeic Acid	OC(=O)/C=C/c1ccc(c(c1)O)O	C9H8O4	No	No	No	No	No	-6.58	0	0	0	0	1	0.56	1	2	1	1.81
Molecule 17	Gallic Acid	OC(=O)c1cc(O)c(c(c1)O)O	C7H6O5	No	No	No	No	Yes	-6.84	0	2	0	0	1	0.56	1	1	1	1.22
Molecule 18	Rhodioloside	OC[C@H]1O[C@@H](OCCc2ccc(cc2)O)[C@@H]([C@H]([C@@H]1O)O)O	C14H20O7	No	No	No	No	No	-8.88	0	1	0	0	0	0.55	0	0	0	4.26
