# Supplementary material for: Xinnaoxin tablets ameliorate high-altitude polycythemia-associated cardiac injury by regulating the NF-κB, MAPK, and PI3K/AKT signaling pathways
Source: Front Pharmacol. 2026 May 28;17:1754806. doi: 10.3389/fphar.2026.1754806 (PMC13253415; doi:10.3389/fphar.2026.1754806)
Supplement: Supplementary file 4 [file DataSheet6.pdf]

	Molecule	smiles	Other ass	Other ass	Blue fluor	Blue fluor	FLuc inhib	FLuc inhib	Promiscuc	Promiscuc	Colloidal a	Colloidal a	Reactive c	Reactive c	Green fluc	Green fluc
0	Sitosterol	C/C=C(\C	0	High	0.003	High	0	High	0	High	0.691	Low	0.001	High	0	High
1	Mandenol	CCCCC/C	0	High	0.004	High	0.011	High	0	High	0.479	Low	0.074	Low	0.001	High
2	Stigmaste	CC[C@@H	0	High	0.004	High	0	High	0.028	Low	0.496	Low	0	High	0	High
3	beta-sitos	CC[C@@H	0	High	0.006	High	0	High	0.01	High	0.571	Low	0	High	0	High
4	atropine	OCC(c1cc	0	High	0.007	High	0	High	0.017	High	0.001	High	0.025	High	0	High
5	glycitein	COc1cc2c	0.865	Low	0.961	Low	0.996	High	0.894	Low	0.323	Low	0	High	0.665	Low
6	7-Dehydr	CC(CCC[C	0	High	0.023	High	0	High	0	High	0.705	Low	0.001	High	0	High
7	7-O-Meth	COc1cc(O	0.885	Low	0.65	Low	0.908	Low	0.95	High	0.888	High	0	High	0.283	Low
8	quercetin	Oc1cc(O)c	0.999	High	0.536	Low	0.809	Low	0.971	Low	0.99	High	0.002	High	0.533	Low
9	sitosterol	CC[C@@H	0	High	0.006	High	0	High	0.01	High	0.571	Low	0	High	0	High
10	pelargoni	Oc1ccc(cc	0.997	High	0.862	Low	0.928	High	0.448	Low	0.956	High	0	High	0.66	Low
11	Beta-caro	C/C(=C\C	0.002	High	0.004	High	0.004	High	0.229	Low	0.395	Low	0	High	0.003	High
12	kaempfer	Oc1ccc(cc	0.999	High	0.497	Low	0.619	Low	0.862	Low	0.968	High	0.001	High	0.583	Low
13	(+)-catech	Oc1cc2O[	0.005	High	0.022	High	0.385	Low	0.421	Low	0.122	Low	0.007	High	0.011	High
14	7-Hydroxy	Oc1ccc2c(	0.966	High	1	High	0.172	Low	0.292	Low	0.69	Low	0	High	0.812	Low
15	Caffeic Ac	OC(=O)/C	0.967	High	0.138	Low	0.985	High	0.972	High	0.255	Low	0.001	High	0.05	High
16	Gallic Acid	OC(=O)c1	0.825	Low	0.001	High	0.321	Low	0.427	Low	0.084	Low	0	High	0.006	High
17	Rhodiolos	OC[C@H]	0	High	0.004	High	0	High	0.032	Low	0	High	0	High	0	High
