# Supplementary material for: Xinnaoxin tablets ameliorate high-altitude polycythemia-associated cardiac injury by regulating the NF-κB, MAPK, and PI3K/AKT signaling pathways
Source: Front Pharmacol. 2026 May 28;17:1754806. doi: 10.3389/fphar.2026.1754806 (PMC13253415; doi:10.3389/fphar.2026.1754806)
Supplement: Supplementary file 5 [file DataSheet9.pdf]

Targets
GSTM1
DNMT1
MB
BCL2
F7
MAPK8
ICAM1
CASP8
NOS3
SELE
NR3C1
ACTB
VCAM1
TNF
JAK1
AKT1
F2
CSNK2B
CTNNB1
MIF
ESR1
ALB
F3
ADRB2
TGFB1
MYC
AR
ESR2
CYP3A4
HSP90AA1
