# Supplementary material for: Xinnaoxin tablets ameliorate high-altitude polycythemia-associated cardiac injury by regulating the NF-κB, MAPK, and PI3K/AKT signaling pathways
Source: Front Pharmacol. 2026 May 28;17:1754806. doi: 10.3389/fphar.2026.1754806 (PMC13253415; doi:10.3389/fphar.2026.1754806)
Supplement: Supplementary file 6 [file DataSheet3.pdf]

	Molecule	Canonical SMILES
0	Sitosterol alpha1	C/C=C(\C(C)C)/CC[C@H]([C@H]1CC[C@@H]2[C@]1(C)CC[C@H]1C2=CC[C@@H]2[C@]1(C)CC[C@@H]([C@H]2C)O)
1	Mandenol	CCCCC/C=C\C/C=C\CCCCCCCC(=O)OCC
2	Stigmasterol	CC[C@@H](C(C)C)/C=C/[C@H]([C@H]1CC[C@@H]2[C@]1(C)CC[C@H]1[C@H]2CC=C2[C@]1(C)CC[C@@H](C2)O)C
3	beta-sitosterol	CC[C@@H](C(C)C)CC[C@H]([C@H]1CC[C@@H]2[C@]1(C)CC[C@H]1[C@H]2CC=C2[C@]1(C)CC[C@@H](C2)O)C
4	atropine	OCC(c1ccccc1)C(=O)O[C@@H]1C[C@@H]2CC[C@H](C1)N2C
5	glycitein	COc1cc2c(cc1O)occ(c2=O)c1ccc(cc1)O
6	7-Dehydrocholesterol	CC(CCC[C@H]([C@H]1CC[C@@H]2[C@]1(C)CC[C@H]1C2=CC=C2[C@]1(C)CC[C@@H](C2)O)C)C
7	7-O-Methylluteolin-6-(	COc1cc(O)c2c(c1)oc(cc2=O)c1ccc(c(c1)O)O
8	quercetin	Oc1cc(O)c2c(c1)oc(c(c2=O)O)c1ccc(c(c1)O)O
9	sitosterol	CC[C@@H](C(C)C)CC[C@H]([C@H]1CC[C@@H]2[C@]1(C)CC[C@H]1[C@H]2CC=C2[C@]1(C)CC[C@@H](C2)O)C
10	pelargonidin	Oc1ccc(cc1)c1[o+]c2cc(O)cc(c2cc1O)O
11	Beta-carotene	C/C(=C\C=C\C=C(\C=C\C=C(\C=C\C1=C(C)CCCC1(C)C)/C)/C)/C=C/C=C(/C=C/C1=C(C)CCCC1(C)C)\C
12	kaempferol	Oc1ccc(cc1)c1oc2cc(O)cc(c2c(=O)c1O)O
13	(+)-catechin	Oc1cc2O[C@H](c3ccc(c(c3)O)O)[C@H](Cc2c(c1)O)O
14	7-Hydroxycoumarin	Oc1ccc2c(c1)oc(=O)cc2
15	Caffeic Acid	OC(=O)/C=C/c1ccc(c(c1)O)O
16	Gallic Acid	OC(=O)c1cc(O)c(c(c1)O)O
17	Rhodioloside	OC[C@H]1O[C@@H](OCCc2ccc(cc2)O)[C@@H]([C@H]([C@@H]1O)O)O
