# Supplementary material for: Xinnaoxin tablets ameliorate high-altitude polycythemia-associated cardiac injury by regulating the NF-κB, MAPK, and PI3K/AKT signaling pathways
Source: Front Pharmacol. 2026 May 28;17:1754806. doi: 10.3389/fphar.2026.1754806 (PMC13253415; doi:10.3389/fphar.2026.1754806)
Supplement: Supplementary file 7 [file DataSheet1.pdf]

Checked	Name	Formula	Annot. Del	Calc. MW	m/z	RT [min]
TRUE	Sucrose	C12 H22 O	-0.75	342.116	341.1086	1.643
TRUE	Kaempferol	C15 H10 O	-0.52	286.0476	285.0402	29.375
TRUE	Betaine	C5 H11 N (	1.25	117.0791	118.0864	1.549
TRUE	Ethyl gallate	C9 H10 O5	-1.09	198.0526	197.0453	22.533
TRUE	α,α-Trehalose	C12 H22 O	-0.75	342.116	341.1086	2.174
TRUE	Gallic acid	C7 H6 O5	-1.23	170.0213	169.014	7.197
TRUE	Salidroside	C14 H20 O	-0.96	300.1206	345.1188	19.352
TRUE	1,2,3,4,6-Pentagalloylglucose 1,2,3,4,(	C41 H32 O	-0.52	940.1177	469.0515	23.145
TRUE	Pyrogallol	C6 H6 O3	-1.9	126.0315	125.0242	8.401
TRUE	Quercetin 7-rhamnoside	C21 H20 O	-0.04	448.1005	449.1083	25.382
TRUE	Rutin	C27 H30 O	0.19	610.1535	611.1614	24.798
TRUE	Choline	C5 H13 N (	1.67	103.0999	104.1072	1.493
TRUE	Gluconic acid	C6 H12 O7	-1.25	196.0581	195.0508	1.539
TRUE	Citric acid	C6 H8 O7	-0.54	192.0269	191.0196	1.804
TRUE	Luteolin	C15 H10 O	-0.41	286.0476	285.0402	26.947
TRUE	Quercetin	C15 H10 O	-0.62	302.0425	301.0351	26.336
TRUE	L-(-)-Malic acid	C4 H6 O5	-1.44	134.0213	133.0141	1.67
TRUE	1,2,3,6-Tetra-O-galloyl-β-D-glucose	C34 H28 O	-0.17	788.1071	787.0998	22.32
TRUE	(-)-Epicatechin gallate	C22 H18 O	0.01	442.09	441.0827	22.906
TRUE	Octyl gallate	C15 H22 O	-1.31	282.1464	281.1391	37.606
TRUE	Afzelin	C21 H20 O	-0.39	432.1055	431.0978	26.587
TRUE	Ellagic acid	C14 H6 O8	-0.77	302.006	300.9988	22.793
TRUE	D-(+)-Galactose	C6 H12 O6	-0.96	180.0632	179.0559	1.579
TRUE	Quinic acid	C7 H12 O6	-2.29	192.063	191.0559	1.612
TRUE	Cannabichromevarin	C19 H26 O	0.55	286.1934	287.2007	47.431
TRUE	1,6-Bis-O-(3,4,5-trihydroxybenzoyl)he	C20 H20 O	-0.22	484.0852	483.0779	19.628
TRUE	4-Oxoproline	C5 H7 N O.	0.47	129.0427	128.0352	4.027
TRUE	4-Aminophenol	C6 H7 N O	2.06	109.053	110.0603	3.703
TRUE	Eriodictyol	C15 H12 O	-0.65	288.0632	287.0558	26.698
TRUE	Miquelianin	C21 H18 O	0.27	478.0749	477.0673	25.155
TRUE	Maltol	C6 H6 O3	2.18	126.032	127.0393	18.649
TRUE	2-Furoic acid	C5 H4 O3	-2.05	112.0158	111.0085	1.769
TRUE	trans-Aconitic acid	C6 H6 O6	-1.7	174.0161	173.0089	1.698
TRUE	Ferulic acid	C10 H10 O	-0.96	194.0577	193.0504	23.108
TRUE	5-Hydroxymethyl-2-furaldehyde	C6 H6 O3	1.83	126.0319	127.0393	1.643
TRUE	Acetophenone	C8 H8 O	0.02	120.0575	121.065	19.315
TRUE	Erucamide	C22 H43 N	0.44	337.3346	338.3419	48.397
TRUE	4-Dodecylbenzenesulfonic acid	C18 H30 O	-0.45	326.1914	325.1841	48.559
TRUE	Demethylwedelolactone	C15 H8 O7	-1.11	300.0267	299.0194	32.44
TRUE	(15Z)-9,12,13-Trihydroxy-15-octadece	C18 H34 O	-0.49	330.2405	329.2332	32.297
TRUE	Catechin	C15 H14 O	-0.19	290.079	289.0717	21.119
TRUE	Salsolinol	C10 H13 N	1.75	179.0949	180.1022	14.406
TRUE	Trigonelline	C7 H7 N O.	1.39	137.0479	138.0552	1.613
TRUE	5,7-Dihydroxychromone	C9 H6 O4	-0.34	178.0266	177.0191	24.241
TRUE	Bioside	C20 H30 O	-0.31	462.1736	461.1664	19.58
TRUE	Kaempferol-7-O-β-D-glucopyranoside	C21 H20 O	-0.06	448.1005	447.0929	23.879
TRUE	Corchorifatty acid F	C18 H32 O	-0.33	328.2249	327.2176	28.843
TRUE	1,2,3-cyclopropanetricarboxylic acid	C6 H6 O6	-1.7	174.0161	173.0089	2.643
TRUE	Pyridoxal	C8 H9 N O.	1.93	167.0586	168.0658	3.657

TRUE	4-Vinylphenol	C8 H8 O	-2.21	120.0573	119.05	22.601
TRUE	Kojic acid	C6 H6 O4	1.86	142.0269	143.0342	18.584
TRUE	2-Isopropylmalic acid	C7 H12 O5	-1.44	176.0682	175.0609	19.922
TRUE	Azelaic acid	C9 H16 O4	-0.66	188.1047	187.0975	24.533
TRUE	2,5-Dihydroxybenzaldehyde	C7 H6 O3	-1.46	138.0315	137.0242	19.657
TRUE	Adenosine	C10 H13 N	-4.07	267.0957	268.1029	12.162
TRUE	Quercetin-3β-D-glucoside	C21 H20 O	0.29	464.0956	463.0881	24.99
TRUE	2-Methylbenzoic acid	C8 H8 O2	-0.73	136.0523	135.045	20.971
TRUE	Astragalin	C21 H20 O	-0.8	448.1002	447.0929	23.677
TRUE	Bilobalide	C15 H18 O	-0.6	326.1	325.0927	20.268
TRUE	3-Hydroxy-2-methylpyridine	C6 H7 N O	2.06	109.053	110.0603	1.65
TRUE	1,2,3,4-Tetrakis-O-(3,4,5-trihydroxybe	C34 H28 O	-0.15	788.1071	787.0998	21.436
TRUE	Lariciresinol 4-O-glucoside	C26 H34 O	-0.72	522.2097	521.2025	22.581
TRUE	Nicotinic acid	C6 H5 N O	2.06	123.0323	124.0396	2.922
TRUE	Citral	C10 H16 O	1.43	152.1203	153.1276	19.991
TRUE	Esculetin	C9 H6 O4	-0.84	178.0265	177.0191	20.93
TRUE	Isorhamnetin	C16 H12 O	0.36	316.0584	315.051	29.768
TRUE	Benzoic acid	C7 H6 O2	1.09	122.0369	123.0442	22.903
TRUE	2,3-Dihydroxybenzoic acid	C7 H6 O4	-1.08	154.0264	153.0192	18.031
TRUE	Myristyl sulfate	C14 H30 O	-0.12	294.1865	293.1792	48.236
TRUE	13(S)-HOTrE	C18 H30 O	0.75	294.2197	295.2271	43.592
TRUE	9-Oxo-ODE	C18 H30 O	0.86	294.2198	295.2271	43.935
TRUE	3-Coumaric acid	C9 H8 O3	-0.71	164.0472	163.0399	20.252
TRUE	Fumaric acid	C4 H4 O4	-2.39	116.0107	115.0034	4.27
TRUE	Gentiopicrin	C16 H20 O	-0.79	356.1105	355.1032	19.761
TRUE	4-Hydroxyphenylacetic acid	C8 H8 O3	-1.17	152.0472	151.0399	20.789
TRUE	Salvianolic acid A	C26 H22 O	-0.07	494.1213	493.114	26.066
TRUE	Shikimic acid	C7 H10 O5	0.46	174.0529	175.0604	1.589
TRUE	2-Hydroxycaproic acid	C6 H12 O3	-1.4	132.0785	131.0712	21.312
TRUE	3-Phenyllactic acid	C9 H10 O3	-1.36	166.0628	165.0555	22.755
TRUE	Methyl gallate	C8 H8 O5	-1.19	184.037	183.0297	20.278
TRUE	(±)9(10)-DiHOME	C18 H34 O	-1.02	314.2454	313.2381	37.683
TRUE	Hyperoside	C21 H20 O	0.71	464.0958	465.1034	22.807
TRUE	5-Phenylnicotinic acid	C12 H9 N (	1.21	199.0636	200.0709	26.973
TRUE	Neochlorogenic acid	C16 H18 O	-0.88	354.0948	353.0875	20.293
TRUE	3,5-Dimethoxy-4-hydroxybenzaldehyc	C9 H10 O4	0.08	182.0579	181.0505	27.898
TRUE	Naringenin	C15 H12 O	-0.48	272.0683	271.0609	28.887
TRUE	Procyanidin B2	C30 H26 O	0.74	578.1429	577.1352	20.636
TRUE	Linoleoyl Ethanolamide	C20 H37 N	0.63	323.2826	324.2899	45.363
TRUE	Terephthalic acid	C8 H6 O4	-1.65	166.0263	165.0191	19.728
TRUE	(+/-)12(13)-DiHOME	C18 H34 O	-0.63	314.2455	313.2381	37.275
TRUE	Uridine	C9 H12 N2	-1.26	244.0692	243.062	6.158
TRUE	Methyl vanillate	C9 H10 O4	0.61	182.058	181.0505	23.014
TRUE	Octyl glucoside	C14 H28 O	0.09	292.1886	293.1959	26.636
TRUE	Trifolin	C21 H20 O	0.14	448.1006	449.1083	22.542
TRUE	D-Raffinose	C18 H32 O	1.92	504.17	527.1592	2.178
TRUE	DL-Tryptophan	C11 H12 N	0.01	204.0899	203.0825	19.068
TRUE	Catechol	C6 H6 O2	-2.29	110.0365	109.0293	18.032
TRUE	Phellamurin	C26 H30 O	0.19	518.1789	517.1713	26.999
TRUE	4-oxo-5-phenylpentanoic acid	C11 H12 O	1.6	192.079	193.0862	20.797

TRUE	(+/-)13-HODE	C18 H32 O	-1.11	296.2348	295.2275	42.093
TRUE	Catalpol	C15 H22 O	-0.19	362.1212	361.114	18.407
TRUE	3,4-Dihydroxybenzaldehyde	C7 H6 O3	1.44	138.0319	139.0392	22.902
TRUE	Taxifolin	C15 H12 O	-0.6	304.0581	303.0508	23.45
TRUE	Cynaroside	C21 H20 O	0.07	448.1006	449.1083	22.904
TRUE	3-oxoindane-1-carboxylic acid	C10 H8 O3	1.42	176.0476	177.0549	49.79
TRUE	Diosmetin	C16 H12 O	-0.19	300.0633	301.0707	29.445
TRUE	L-Phenylalanine	C9 H11 N (	-0.54	165.0789	164.0714	17.985
TRUE	trans-Cinnamaldehyde	C9 H8 O	1.25	132.0577	133.065	20.42
TRUE	D-(-)-Lyxose	C5 H10 O5	-2.13	150.0525	149.0452	2.535
TRUE	Tetrahydroxyxanthone	C13 H8 O6	-1.29	260.0318	259.0245	23.516
TRUE	ethyl 5-hydroxy-4-oxo-4H-chromene-2	C12 H10 O	1.42	234.0532	235.0604	23.834
TRUE	3-Methoxybenzaldehyde	C8 H8 O2	1.86	136.0527	137.0599	18.414
TRUE	2-[2-(2-pyridyloxy)ethoxy]pyridine	C12 H12 N	1.76	216.0903	217.0975	18.211
TRUE	Phenacetin	C10 H13 N	1.79	179.095	180.1022	19.797
TRUE	Baicalin	C21 H18 O	1.45	446.0856	447.0928	25.198
TRUE	2-Ethylamino-1-phenylpropanol	C11 H17 N	1.7	179.1313	180.1386	19.448
TRUE	Gentisic acid	C7 H6 O4	-1.08	154.0264	153.0192	19.991
TRUE	Daidzein	C15 H10 O	0.54	254.0581	255.0653	26.229
TRUE	Rosmarinic acid	C18 H16 O	0.17	360.0846	359.0773	24.453
TRUE	Neochlorogenic acid	C16 H18 O	-0.88	354.0948	353.0875	19.103
TRUE	2-(2-acetyl-3,5-dihydroxyphenyl)aceti	C10 H10 O	0.89	210.053	211.0603	32.191
TRUE	Dimethyl Phthalate	C10 H10 O	-1.12	194.0577	193.0504	22.544
TRUE	Fisetin	C15 H10 O	0.46	286.0479	287.0552	22.537
TRUE	4-Hydroxybenzoic acid	C7 H6 O3	1.44	138.0319	139.0392	21.128
TRUE	Desoxyrhaponticin	C21 H24 O	-0.7	404.1468	403.1396	25.491
TRUE	Triphenyl phosphate	C18 H15 O	0.44	326.0709	327.0782	62.963
TRUE	trans-3-Indoleacrylic acid	C11 H9 N (	1.94	187.0637	188.071	19.174
TRUE	4-Pyridineacetic acid	C7 H7 N O	0.12	137.0477	138.0552	19.982
TRUE	N6-Isopentenyladenosine	C15 H21 N	0.53	335.1595	336.1668	22.829
TRUE	Adenosine 5'-monophosphate	C10 H14 N	0.6	347.0633	348.0707	3.569
TRUE	Cycloolivil	C20 H24 O	-0.23	376.1521	375.1448	21.76
TRUE	(-)-Fustin	C15 H12 O	0.68	288.0636	289.0709	22.449
TRUE	12-oxo Phytodienoic Acid	C18 H28 O	0.94	292.2041	293.2114	35.039
TRUE	3,4-Dihydroxyphenylethanol	C8 H10 O3	-1.14	154.0628	153.0555	18.535
TRUE	D-(+)-Camphor	C10 H16 O	1.43	152.1203	153.1276	50.211
TRUE	Bergaptol	C11 H6 O4	-0.6	202.0265	201.0192	27.161
TRUE	Rebaudioside A	C44 H70 O	-0.29	966.4305	965.4232	27.268
TRUE	Bengenin	C14 H16 O	-1.15	328.0791	327.0718	19.608
TRUE	Safrole	C10 H10 O	1.46	162.0683	163.0756	20.913
TRUE	Galangin	C15 H10 O	0.75	270.053	271.0603	21.945
TRUE	N-Acetylvaline	C7 H13 N (	-1.38	159.0893	158.0821	19.162
TRUE	trans-Cinnamaldehyde	C9 H8 O	1.25	132.0577	133.065	25.969
TRUE	2-Methoxybenzaldehyde	C8 H8 O2	1.47	136.0526	137.0599	19.418
TRUE	2-Anisic acid	C8 H8 O3	-1.39	152.0471	151.0399	22.066
TRUE	4-hydroxy-5,8-dimethylquinoline-3-ca	C12 H11 N	1.3	217.0742	218.0815	24.993
TRUE	Heptanophenone	C13 H18 O	1.41	190.136	191.1433	35.484
TRUE	Daidzin	C21 H20 O	1.25	416.1113	417.1185	21.754
TRUE	Myricetin	C15 H10 O	0.92	318.0379	319.0451	23.958
TRUE	Medicarpin	C16 H14 O	-0.24	270.0891	269.0818	35.677

TRUE	1,2,3,4-Tetramethyl-1,3-cyclopentadi	C9 H14	1.76	122.1098	123.117	26.84
TRUE	4-oxododecanedioic acid	C12 H20 O	0.75	244.1313	245.1385	23.655
TRUE	4-(allyloxy)-1,2-dihydroquinolin-2-one	C12 H11 N	1.38	201.0793	202.0865	26.445
TRUE	N,N-Dimethyldecylamine N-oxide	C12 H27 N	1.16	201.2095	202.2168	28.993
TRUE	Emodin	C15 H10 O	-0.62	270.0527	269.0454	37.888
TRUE	7-Demethylsuberosin	C14 H14 O	1.02	230.0945	231.1018	24.426
TRUE	4-methoxy-6-(prop-2-en-1-yl)-2H-1,3-	C11 H12 O	1.53	192.0789	193.0862	23.37
TRUE	(+)-Pinoresinol	C20 H22 O	-0.06	358.1416	357.1343	23.402
TRUE	α-Linolenoyl ethanolamide	C20 H35 N	1.21	321.2672	322.2744	43.456
TRUE	Berberine	C20 H17 N	0.77	335.116	336.1233	26.449
TRUE	4-hydroxy-3-(3-methylbut-2-en-1-yl)b	C12 H14 O	1.29	206.0946	207.1018	28.585
TRUE	Psoralen	C11 H6 O3	1.64	186.032	187.0393	27.282
TRUE	Guanosine	C10 H13 N	-0.47	283.0915	282.0843	14.77
TRUE	(+/-)-CP 47,497-C7-Hydroxy metabolit	C21 H34 O	0.69	334.251	335.2583	38.36
TRUE	α-Linolenic acid	C18 H30 O	1.04	278.2249	279.2322	44.787
TRUE	Eugenol	C10 H12 O	1.84	164.084	165.0913	24.759
TRUE	Orcinol glucoside	C13 H18 O	-0.9	286.105	285.0977	13.097
TRUE	cis-2-Decenoic acid	C10 H18 O	-1.47	170.1304	169.1232	31.802
TRUE	(±)9-HpODE	C18 H32 O	-0.7	312.2298	311.2226	40.249
TRUE	Citrinin	C13 H14 O	0.37	250.0842	251.0915	32.477
TRUE	8-Prenylnaringenin	C20 H20 O	-0.54	340.1309	339.1234	38.49
TRUE	Cinnamic acid	C9 H8 O2	1.76	148.0527	149.06	29.935
TRUE	Rheic acid	C15 H8 O6	-0.43	284.032	283.0247	33.274
TRUE	Testosterone 17-Undecanoate	C30 H48 O	-0.29	456.3602	455.3529	52.537
TRUE	Hexadecanedioic acid	C16 H30 O	-0.57	286.2143	285.207	39.545
TRUE	Shogaol	C17 H24 O	1.17	276.1729	277.1802	34.019
TRUE	Methyl cinnamate	C10 H10 O	1.46	162.0683	163.0756	26.489

mzCloud B	mzVault B	MS2	Reference	Group Area: W-3
94.7	93.8	DDA for pr	[M-H]-1	1.56E+10
85.9	93.8	DDA for pr	[M-H]-1	1.29E+10
97.1	94.2	DDA for pr	[M+H]+1	9.9E+09
	92.6	DDA for pr	[M-H]-1	7.39E+09
94.9	95.1	DDA for pr	[M-H]-1	7.04E+09
91	91.9	DDA for pr	[M-H]-1	6.69E+09
	92	DDA for pr	[M+FA-H]-	4.58E+09
	91.8	DDA for pr	[M-2H]-2	4.5E+09
88.8	90.8	DDA for pr	[M-H]-1	4.37E+09
80.7	79.3	DDA for pr	[M+H]+1	4.26E+09
87.7	84.1	DDA for pr	[M+H]+1	4.25E+09
95.6	96.3	DDA for pr	[M+H]+1	3.8E+09
92.5	94.5	DDA for pr	[M-H]-1	3.68E+09
88	95.4	DDA for pr	[M-H]-1	3.33E+09
83.2	94.4	DDA for pr	[M-H]-1	3.22E+09
79.6	66.6	DDA for pr	[M-H]-1	3.22E+09
93.2	97.6	DDA for pr	[M-H]-1	2.85E+09
88.6	85.4	DDA for pr	[M-H]-1	2.6E+09
	92.2	DDA for pr	[M-H]-1	2.55E+09
90.1	92.8	DDA for pr	[M-H]-1	2.5E+09
83.5	83.3	DDA for pr	[M-H]-1	1.94E+09
	90	DDA for pr	[M-H]-1	1.94E+09
81	82.9	DDA for pr	[M-H]-1	1.35E+09
84.4	82.7	DDA for pr	[M-H]-1	1.23E+09
75.3	56.5	DDA for pr	[M+H]+1	1.12E+09
91.3	91	DDA for pr	[M-H]-1	9.66E+08
94.2	86.8	DDA for pr	[M-H]-1	9.43E+08
81.5	86.5	DDA for pr	[M+H]+1	8.47E+08
86	90	DDA for pr	[M-H]-1	7.77E+08
79.7	79.7	DDA for pr	[M-H]-1	7.26E+08
88.6	91.7	DDA for pr	[M+H]+1	7.09E+08
92.6	85.3	DDA for pr	[M-H]-1	6.79E+08
83.7	88.4	DDA for pr	[M-H]-1	5.92E+08
83.4	69.1	DDA for pr	[M-H]-1	5.52E+08
82	87.2	DDA for pr	[M+H]+1	5.52E+08
85.3	89	DDA for pr	[M+H]+1	5.48E+08
94	90.8	DDA for pr	[M+H]+1	5.09E+08
90.3	91.3	DDA for pr	[M-H]-1	4.86E+08
	78.6	DDA for pr	[M-H]-1	4.79E+08
77.7	79.4	DDA for pr	[M-H]-1	4.62E+08
88.8	91.5	DDA for pr	[M-H]-1	4.59E+08
71.3	58.8	DDA for pr	[M+H]+1	4.58E+08
89.7	80.2	DDA for pr	[M+H]+1	4.23E+08
	91.4	DDA for pr	[M-H]-1	3.19E+08
71.5	70.6	DDA for pr	[M-H]-1	3.12E+08
84.2	89.9	DDA for pr	[M-H]-1	3E+08
90.3	87.7	DDA for pr	[M-H]-1	2.93E+08
84.3	88	DDA for pr	[M-H]-1	2.79E+08
76.4	66.4	DDA for pr	[M+H]+1	2.7E+08

85.4	73.8	DDA for pr	[M-H]-1	2.67E+08
80.3	67.4	DDA for pr	[M+H]+1	2.57E+08
94.4	96.3	DDA for pr	[M-H]-1	2.37E+08
93.2	95.5	DDA for pr	[M-H]-1	2.35E+08
88.9	86.7	DDA for pr	[M-H]-1	2.27E+08
84.6	83.5	DDA for pr	[M+H]+1	2.25E+08
81.6	84.4	DDA for pr	[M-H]-1	2.19E+08
72.9	57.8	DDA for pr	[M-H]-1	2.13E+08
85.8	88.8	DDA for pr	[M-H]-1	2.07E+08
	70.8	DDA for pr	[M-H]-1	2.05E+08
68.7	79.4	DDA for pr	[M+H]+1	2.04E+08
86	84.2	DDA for pr	[M-H]-1	1.94E+08
88.3	84.4	DDA for pr	[M-H]-1	1.9E+08
82.5	81.9	DDA for pr	[M+H]+1	1.83E+08
63.6	73.1	DDA for pr	[M+H]+1	1.8E+08
	81.8	DDA for pr	[M-H]-1	1.6E+08
85.8	95.7	DDA for pr	[M-H]-1	1.58E+08
85.5	90.3	DDA for pr	[M+H]+1	1.57E+08
93.1	93.1	DDA for pr	[M-H]-1	1.51E+08
83.2	78.3	DDA for pr	[M-H]-1	1.31E+08
81.6	81.4	DDA for pr	[M+H]+1	1.3E+08
91.3	81.5	DDA for pr	[M+H]+1	1.21E+08
73.4	86.2	DDA for pr	[M-H]-1	1.21E+08
	73.4	DDA for pr	[M-H]-1	1.07E+08
	76.5	DDA for pr	[M-H]-1	1.07E+08
87.3	83.2	DDA for pr	[M-H]-1	1.06E+08
	82.9	DDA for pr	[M-H]-1	1.03E+08
	70	DDA for pr	[M+H]+1	1.03E+08
80.9	92.2	DDA for pr	[M-H]-1	1.03E+08
79.5	86.4	DDA for pr	[M-H]-1	1.03E+08
	79.9	DDA for pr	[M-H]-1	1.01E+08
80.9	87.6	DDA for pr	[M-H]-1	95328231
85.3	87.6	DDA for pr	[M+H]+1	93088096
76.2	69.4	DDA for pr	[M+H]+1	89797968
85	90.5	DDA for pr	[M-H]-1	89216704
77	75.8	DDA for pr	[M-H]-1	89033326
86.8	92.1	DDA for pr	[M-H]-1	83204441
	87.2	DDA for pr	[M-H]-1	83070797
88.6	84.5	DDA for pr	[M+H]+1	79637885
81.4	81.5	DDA for pr	[M-H]-1	77175615
75.9	87.4	DDA for pr	[M-H]-1	74142463
82.8	89.7	DDA for pr	[M-H]-1	64711641
71.3	72.3	DDA for pr	[M-H]-1	63264634
79.7	80.1	DDA for pr	[M+H]+1	62983241
83.5	83	DDA for pr	[M+H]+1	59995096
83.2	83.1	DDA for ot	[M+Na]+1	55928015
84.2	86.8	DDA for pr	[M-H]-1	54418624
81.8	78.5	DDA for pr	[M-H]-1	54343576
	84.9	DDA for pr	[M-H]-1	54200835
66.1	78.1	DDA for pr	[M+H]+1	53126182

	79.2	DDA for pr	[M-H]-1	51210273
	71.9	DDA for pr	[M-H]-1	51209767
75.1	54.4	DDA for pr	[M+H]+1	51028584
	84.8	DDA for pr	[M-H]-1	50706410
84.4	83.4	DDA for pr	[M+H]+1	48660465
77.4	73.7	DDA for pr	[M+H]+1	45788835
77.6	79.5	DDA for pr	[M+H]+1	45550849
64.9	81.4	DDA for pr	[M-H]-1	42726804
84.5	87.6	DDA for pr	[M+H]+1	41470986
66	81.1	DDA for pr	[M-H]-1	41013341
71.6	68.4	DDA for pr	[M-H]-1	40816328
	71.5	DDA for pr	[M+H]+1	39815556
70.6	76.7	DDA for pr	[M+H]+1	38564072
	76.2	DDA for pr	[M+H]+1	38498635
70.8		DDA for pr	[M+H]+1	37691800
80.4	82.4	DDA for pr	[M+H]+1	36971287
70.5	68.2	DDA for pr	[M+H]+1	36521325
76.4	79.6	DDA for pr	[M-H]-1	36313220
87.2	88.1	DDA for pr	[M+H]+1	35880292
62	78.2	DDA for pr	[M-H]-1	34428509
70	68.5	DDA for pr	[M-H]-1	34283151
	79.9	DDA for pr	[M+H]+1	34055666
77.6		DDA for pr	[M-H]-1	33173900
75	66.4	DDA for pr	[M+H]+1	32398274
74.4	59.8	DDA for pr	[M+H]+1	31954005
	81.3	DDA for pr	[M-H]-1	31864261
62	85.9	DDA for pr	[M+H]+1	31701753
85.9	87	DDA for pr	[M+H]+1	31667574
73	77.7	DDA for pr	[M+H]+1	31414048
	87.5	DDA for pr	[M+H]+1	31303627
82.8	83.5	DDA for pr	[M+H]+1	30370619
75.4	83.4	DDA for pr	[M-H]-1	30047215
75.9	54.5	DDA for pr	[M+H]+1	29697621
81.3	80.5	DDA for pr	[M+H]+1	29535796
69.8	73.2	DDA for pr	[M-H]-1	28763269
73.7	85.3	DDA for pr	[M+H]+1	27768757
	84.3	DDA for pr	[M-H]-1	27499113
	79.9	DDA for pr	[M-H]-1	27199399
	73.4	DDA for pr	[M-H]-1	27188741
	70.4	DDA for pr	[M+H]+1	27134155
79.5	69	DDA for pr	[M+H]+1	27093190
81.5	83.1	DDA for pr	[M-H]-1	27037739
77.6	80.1	DDA for pr	[M+H]+1	26535743
60.4	71.9	DDA for pr	[M+H]+1	25374993
87.3	86.1	DDA for pr	[M-H]-1	24704994
68	71.7	DDA for pr	[M+H]+1	22973107
73.5	56.6	DDA for pr	[M+H]+1	22170150
74.3	80.8	DDA for pr	[M+H]+1	21939238
82.3	62.8	DDA for pr	[M+H]+1	20785495
71.4	80.4	DDA for pr	[M-H]-1	20468662

75.4	87.4	DDA for pr	[M+H]+1	19518235
	71.1	DDA for pr	[M+H]+1	18694327
	72.2	DDA for pr	[M+H]+1	18289338
79.1	53.1	DDA for pr	[M+H]+1	18024232
86	77.6	DDA for pr	[M-H]-1	17784034
	72.5	DDA for pr	[M+H]+1	16016721
	76.5	DDA for pr	[M+H]+1	14544105
	82.7	DDA for pr	[M-H]-1	14100549
74.9	64.4	DDA for pr	[M+H]+1	14034045
92.4	89.4	DDA for pr	[M+H]+1	13585117
60.9	83.6	DDA for pr	[M+H]+1	12217416
82.7	81.9	DDA for pr	[M+H]+1	11797257
85.3	93.3	DDA for pr	[M-H]-1	10778841
	77.6	DDA for pr	[M+H]+1	10510582
68.2	73.8	DDA for pr	[M+H]+1	10408400
69	73.8	DDA for pr	[M+H]+1	10397495
	71.3	DDA for pr	[M-H]-1	9689594
	72.6	DDA for pr	[M-H]-1	9621475
76.4	74.6	DDA for pr	[M-H]-1	8545100
64.9	70.3	DDA for pr	[M+H]+1	8238126
	85.5	DDA for pr	[M-H]-1	7867997
	79.3	DDA for pr	[M+H]+1	7726592
	84.4	DDA for pr	[M-H]-1	7298181
88.2		DDA for pr	[M-H]-1	6731582
65.8	84.6	DDA for pr	[M-H]-1	4801604
66.1	84.5	DDA for pr	[M+H]+1	3680332
	72.2	DDA for pr	[M+H]+1	3463539
