# Supplementary material for: Xinnaoxin tablets ameliorate high-altitude polycythemia-associated cardiac injury by regulating the NF-κB, MAPK, and PI3K/AKT signaling pathways
Source: Front Pharmacol. 2026 May 28;17:1754806. doi: 10.3389/fphar.2026.1754806 (PMC13253415; doi:10.3389/fphar.2026.1754806)
Supplement: Supplementary file 8 [file DataSheet5.pdf]

Molecule 1

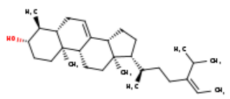

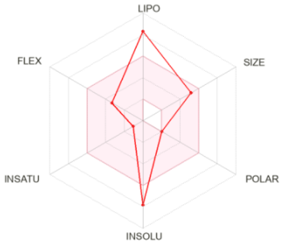

SMILES C/C=C(\C(C)C)/CC[C@H]([C@H]1CC[C@@H]2[C@]1(C)CC[C@H]1C2=CC[C@@H]2[C@]1(C)CC[C@@H]([C@H]2C)O)C

Physicochemical Properties	
Formula	C30H50O
Molecular weight	426.72 g/mol
Num. heavy atoms	31
Num. arom. heavy atoms	0
Fraction Csp3	0.87
Num. rotatable bonds	5
Num. H-bond acceptors	1
Num. H-bond donors	1
Molar Refractivity	137.56
TPSA	20.23 Å²

Lipophilicity	
Log $P_{o/w}$ (iLOGP)	5.24
Log $P_{o/w}$ (XLOGP3)	9.03
Log $P_{o/w}$ (WLOGP)	8.19
Log $P_{o/w}$ (MLOGP)	6.82
Log $P_{o/w}$ (SILICOS-IT)	7.00
Consensus Log $P_{o/w}$	7.26

Water Solubility	
Log *S* (ESOL)	-7.84
Solubility	6.10e-06 mg/ml ; 1.43e-08 mol/l
Class	Poorly soluble
Log *S* (Ali)	-9.35
Solubility	1.92e-07 mg/ml ; 4.50e-10 mol/l
Class	Poorly soluble
Log *S* (SILICOS-IT)	-5.97
Solubility	4.57e-04 mg/ml ; 1.07e-06 mol/l
Class	Moderately soluble

Pharmacokinetics	
GI absorption	Low
BBB permeant	No
P-gp substrate	No
CYP1A2 inhibitor	No
CYP2C19 inhibitor	No
CYP2C9 inhibitor	No
CYP2D6 inhibitor	No
CYP3A4 inhibitor	No
Log K_p (skin permeation)	-2.49 cm/s

Druglikeness	
Lipinski	Yes; 1 violation: MLOGP>4.15
Ghose	No; 3 violations: WLOGP>5.6, MR>130, #atoms>70
Veber	Yes
Egan	No; 1 violation: WLOGP>5.88
Muegge	No; 2 violations: XLOGP3>5, Heteroatoms<2
Bioavailability Score	0.55

Medicinal Chemistry	
PAINS	0 alert
Brenk	1 alert: isolated_alkene
Leadlikeness	No; 2 violations: MW>350, XLOGP3>3.5
Synthetic accessibility	6.22

Molecule 2

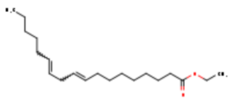

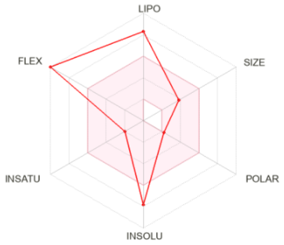

SMILES CCCCC/C=C\C/C=C\CCCCCCCC(=O)OCC

Physicochemical Properties	
Formula	C20H36O2
Molecular weight	308.50 g/mol
Num. heavy atoms	22
Num. arom. heavy atoms	0
Fraction Csp3	0.75
Num. rotatable bonds	16
Num. H-bond acceptors	2
Num. H-bond donors	0
Molar Refractivity	98.59
TPSA	26.30 Å²

Lipophilicity	
Log $P_{o/w}$ (iLOGP)	5.24
Log $P_{o/w}$ (XLOGP3)	9.03
Log $P_{o/w}$ (WLOGP)	6.36
Log $P_{o/w}$ (MLOGP)	6.82
Log $P_{o/w}$ (SILICOS-IT)	7.00
Consensus Log $P_{o/w}$	7.26

Water Solubility	
Log *S* (ESOL)	-7.84
Solubility	6.10e-06 mg/ml ; 1.43e-08 mol/l
Class	Poorly soluble
Log *S* (Ali)	-9.35
Solubility	1.92e-07 mg/ml ; 4.50e-10 mol/l
Class	Poorly soluble
Log *S* (SILICOS-IT)	-5.97
Solubility	4.57e-04 mg/ml ; 1.07e-06 mol/l
Class	Moderately soluble

Pharmacokinetics	
GI absorption	Low
BBB permeant	No
P-gp substrate	No
CYP1A2 inhibitor	No
CYP2C19 inhibitor	No
CYP2C9 inhibitor	No
CYP2D6 inhibitor	No
CYP3A4 inhibitor	No
Log K_p (skin permeation)	-2.49 cm/s

Druglikeness	
Lipinski	Yes; 1 violation: MLOGP>4.15
Ghose	No; 3 violations: WLOGP>5.6, MR>130, #atoms>70
Veber	Yes
Egan	No; 1 violation: WLOGP>5.88
Muegge	No; 2 violations: XLOGP3>5, Heteroatoms<2
Bioavailability Score	0.55

Medicinal Chemistry	
PAINS	0 alert
Brenk	1 alert: isolated_alkene
Leadlikeness	No; 2 violations: MW>350, XLOGP3>3.5
Synthetic accessibility	6.22

Molecule 3

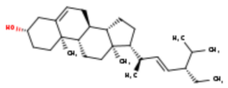

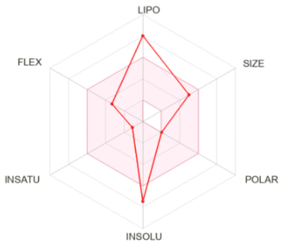

SMILES CC[C@@H](C(C)C)/C=C/[C@H]([C@H]1CC[C@@H]2[C@]1(C)CC[C@H]1[C@H]2CC=C2[C@]1(C)CC[C@@H](C2)O)C

Physicochemical Properties	
Formula	C29H48O
Molecular weight	412.69 g/mol
Num. heavy atoms	30
Num. arom. heavy atoms	0
Fraction Csp3	0.86
Num. rotatable bonds	5
Num. H-bond acceptors	1
Num. H-bond donors	1
Molar Refractivity	132.75
TPSA	20.23 Å²

Lipophilicity	
Log $P_{o/w}$ (iLOGP)	5.08
Log $P_{o/w}$ (XLOGP3)	8.56
Log $P_{o/w}$ (WLOGP)	7.80
Log $P_{o/w}$ (MLOGP)	6.62
Log $P_{o/w}$ (SILICOS-IT)	6.86
Consensus Log $P_{o/w}$	6.98

Water Solubility	
Log *S* (ESOL)	-7.46
Solubility	1.43e-05 mg/ml ; 3.46e-08 mol/l
Class	Poorly soluble
Log *S* (Ali)	-8.86
Solubility	5.71e-07 mg/ml ; 1.38e-09 mol/l
Class	Poorly soluble
Log *S* (SILICOS-IT)	-5.47
Solubility	1.40e-03 mg/ml ; 3.39e-06 mol/l
Class	Moderately soluble

Pharmacokinetics	
GI absorption	Low
BBB permeant	No
P-gp substrate	No
CYP1A2 inhibitor	No
CYP2C19 inhibitor	No
CYP2C9 inhibitor	Yes
CYP2D6 inhibitor	No
CYP3A4 inhibitor	No
Log K_p (skin permeation)	-2.74 cm/s

Druglikeness	
Lipinski	Yes; 1 violation: MLOGP>4.15
Ghose	No; 3 violations: WLOGP>5.6, MR>130, #atoms>70
Veber	Yes
Egan	No; 1 violation: WLOGP>5.88
Muegge	No; 2 violations: XLOGP3>5, Heteroatoms<2
Bioavailability Score	0.55

Medicinal Chemistry	
PAINS	0 alert
Brenk	1 alert: isolated_alkene
Leadlikeness	No; 2 violations: MW>350, XLOGP3>3.5
Synthetic accessibility	6.21

Molecule 4

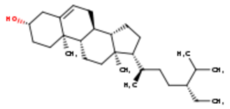

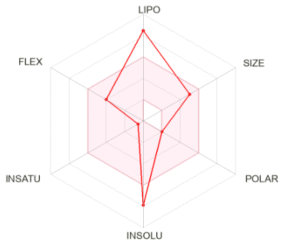

SMILES CC[C@@H](C(C)C)CC[C@H]([C@H]1CC[C@@H]2[C@]1(C)CC[C@H]1[C@H]2CC=C2[C@]1(C)CC[C@@H](C2)O)C

Physicochemical Properties	
Formula	C29H50O
Molecular weight	414.71 g/mol
Num. heavy atoms	30
Num. arom. heavy atoms	0
Fraction Csp3	0.93
Num. rotatable bonds	6
Num. H-bond acceptors	1
Num. H-bond donors	1
Molar Refractivity	133.23
TPSA	20.23 Å²

Lipophilicity	
Log $P_{o/w}$ (iLOGP)	5.05
Log $P_{o/w}$ (XLOGP3)	9.34
Log $P_{o/w}$ (WLOGP)	8.02
Log $P_{o/w}$ (MLOGP)	6.73
Log $P_{o/w}$ (SILICOS-IT)	7.04
Consensus Log $P_{o/w}$	7.24

Water Solubility	
Log *S* (ESOL)	-7.90
Solubility	5.23e-06 mg/ml ; 1.26e-08 mol/l
Class	Poorly soluble
Log *S* (Ali)	-9.67
Solubility	8.90e-08 mg/ml ; 2.15e-10 mol/l
Class	Poorly soluble
Log *S* (SILICOS-IT)	-6.19
Solubility	2.69e-04 mg/ml ; 6.49e-07 mol/l
Class	Poorly soluble

Pharmacokinetics	
GI absorption	Low
BBB permeant	No
P-gp substrate	No
CYP1A2 inhibitor	No
CYP2C19 inhibitor	No
CYP2C9 inhibitor	No
CYP2D6 inhibitor	No
CYP3A4 inhibitor	No
Log K_p (skin permeation)	-2.20 cm/s

Druglikeness	
Lipinski	Yes; 1 violation: MLOGP>4.15
Ghose	No; 3 violations: WLOGP>5.6, MR>130, #atoms>70
Veber	Yes
Egan	No; 1 violation: WLOGP>5.88
Muegge	No; 2 violations: XLOGP3>5, Heteroatoms<2
Bioavailability Score	0.55

Medicinal Chemistry	
PAINS	0 alert
Brenk	1 alert: isolated_alkene
Leadlikeness	No; 2 violations: MW>350, XLOGP3>3.5
Synthetic accessibility	6.30

Molecule 5

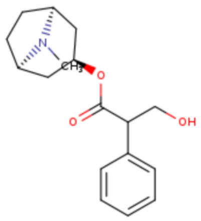

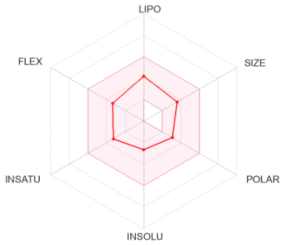

SMILES OCC(c1ccccc1)C(=O)O[C@@H]1C[C@@H]2CC[C@H](C1)N2C

Physicochemical Properties	
Formula	C17H23NO3
Molecular weight	289.37 g/mol
Num. heavy atoms	21
Num. arom. heavy atoms	6
Fraction Csp3	0.59
Num. rotatable bonds	5
Num. H-bond acceptors	4
Num. H-bond donors	1
Molar Refractivity	84.51
TPSA	49.77 Å²

Lipophilicity	
Log $P_{o/w}$ (iLOGP)	2.61
Log $P_{o/w}$ (XLOGP3)	1.83
Log $P_{o/w}$ (WLOGP)	1.55
Log $P_{o/w}$ (MLOGP)	2.02
Log $P_{o/w}$ (SILICOS-IT)	2.03
Consensus Log $P_{o/w}$	2.01

Water Solubility	
Log *S* (ESOL)	-2.67
Solubility	6.21e-01 mg/ml ; 2.15e-03 mol/l
Class	Soluble
Log *S* (Ali)	-2.50
Solubility	9.25e-01 mg/ml ; 3.20e-03 mol/l
Class	Soluble
Log *S* (SILICOS-IT)	-2.93
Solubility	3.41e-01 mg/ml ; 1.18e-03 mol/l
Class	Soluble

Pharmacokinetics	
GI absorption	High
BBB permeant	Yes
P-gp substrate	No
CYP1A2 inhibitor	No
CYP2C19 inhibitor	No
CYP2C9 inhibitor	No
CYP2D6 inhibitor	Yes
CYP3A4 inhibitor	No
Log K_p (skin permeation)	-6.77 cm/s

Druglikeness	
Lipinski	Yes; 0 violation
Ghose	Yes
Veber	Yes
Egan	Yes
Muegge	Yes
Bioavailability Score	0.55

Medicinal Chemistry	
PAINS	0 alert
Brenk	0 alert
Leadlikeness	Yes
Synthetic accessibility	4.33

Molecule 6

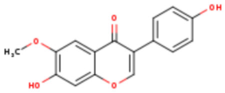

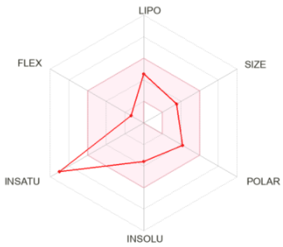

SMILES COc1cc2c(cc1O)occ(c2=O)c1ccc(cc1)O

Physicochemical Properties	
Formula	C16H12O5
Molecular weight	284.26 g/mol
Num. heavy atoms	21
Num. arom. heavy atoms	16
Fraction Csp3	0.06
Num. rotatable bonds	2
Num. H-bond acceptors	5
Num. H-bond donors	2
Molar Refractivity	78.46
TPSA	79.90 Å²

Lipophilicity	
Log $P_{o/w}$ (iLOGP)	2.36
Log $P_{o/w}$ (XLOGP3)	2.44
Log $P_{o/w}$ (WLOGP)	2.88
Log $P_{o/w}$ (MLOGP)	0.77
Log $P_{o/w}$ (SILICOS-IT)	3.03
Consensus Log $P_{o/w}$	2.30

Water Solubility	
Log *S* (ESOL)	-3.57
Solubility	7.63e-02 mg/ml ; 2.68e-04 mol/l
Class	Soluble
Log *S* (Ali)	-3.76
Solubility	4.93e-02 mg/ml ; 1.73e-04 mol/l
Class	Soluble
Log *S* (SILICOS-IT)	-5.10
Solubility	2.25e-03 mg/ml ; 7.91e-06 mol/l
Class	Moderately soluble

Pharmacokinetics	
GI absorption	High
BBB permeant	No
P-gp substrate	No
CYP1A2 inhibitor	Yes
CYP2C19 inhibitor	No
CYP2C9 inhibitor	No
CYP2D6 inhibitor	Yes
CYP3A4 inhibitor	Yes
Log K_p (skin permeation)	-6.30 cm/s

Druglikeness	
Lipinski	Yes; 0 violation
Ghose	Yes
Veber	Yes
Egan	Yes
Muegge	Yes
Bioavailability Score	0.55

Medicinal Chemistry	
PAINS	0 alert
Brenk	0 alert
Leadlikeness	Yes
Synthetic accessibility	2.95

Molecule 7

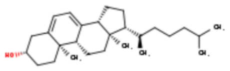

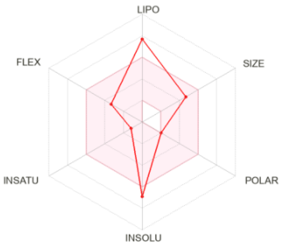

SMILES CC(CCC[C@H]([C@H]1CC[C@@H]2[C@]1(C)CC[C@H]1C2=CC=C2[C@]1(C)CC[C@@H](C2)O)C)C

Physicochemical Properties	
Formula	C27H44O
Molecular weight	384.64 g/mol
Num. heavy atoms	28
Num. arom. heavy atoms	0
Fraction Csp3	0.85
Num. rotatable bonds	5
Num. H-bond acceptors	1
Num. H-bond donors	1
Molar Refractivity	123.14
TPSA	20.23 Å²

Lipophilicity	
Log $P_{o/w}$ (iLOGP)	4.90
Log $P_{o/w}$ (XLOGP3)	7.96
Log $P_{o/w}$ (WLOGP)	7.31
Log $P_{o/w}$ (MLOGP)	6.23
Log $P_{o/w}$ (SILICOS-IT)	6.39
Consensus Log $P_{o/w}$	6.56

Water Solubility	
Log S (ESOL)	-6.91
Solubility	4.74e-05 mg/ml ; 1.23e-07 mol/l
Class	Poorly soluble
Log S (Ali)	-8.24
Solubility	2.23e-06 mg/ml ; 5.81e-09 mol/l
Class	Poorly soluble
Log S (SILICOS-IT)	-5.76
Solubility	6.70e-04 mg/ml ; 1.74e-06 mol/l
Class	Moderately soluble

Pharmacokinetics	
GI absorption	Low
BBB permeant	No
P-gp substrate	No
CYP1A2 inhibitor	No
CYP2C19 inhibitor	No
CYP2C9 inhibitor	Yes
CYP2D6 inhibitor	No
CYP3A4 inhibitor	No
Log K_p (skin permeation)	-2.99 cm/s

Druglikeness	
Lipinski	Yes; 1 violation: MLOGP>4.15
Ghose	No; 2 violations: WLOGP>5.6, #atoms>70
Veber	Yes
Egan	No; 1 violation: WLOGP>5.88
Muegge	No; 2 violations: XLOGP3>5, Heteroatoms<2
Bioavailability Score	0.55

Medicinal Chemistry	
PAINS	0 alert
Brenk	0 alert
Leadlikeness	No; 2 violations: MW>350, XLOGP3>3.5
Synthetic accessibility	6.45

Molecule 8

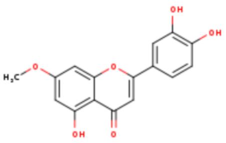

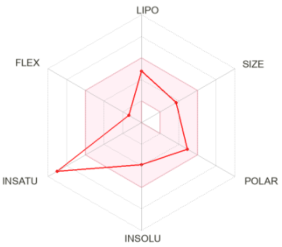

SMILES COc1cc(O)c2c(c1)oc(cc2=O)c1ccc(c(c1)O)O

Physicochemical Properties	
Formula	C16H12O6
Molecular weight	300.26 g/mol
Num. heavy atoms	22
Num. arom. heavy atoms	16
Fraction Csp3	0.06
Num. rotatable bonds	2
Num. H-bond acceptors	6
Num. H-bond donors	3
Molar Refractivity	80.48
TPSA	100.13 Å²

Lipophilicity	
Log $P_{o/w}$ (iLOGP)	2.45
Log $P_{o/w}$ (XLOGP3)	2.86
Log $P_{o/w}$ (WLOGP)	2.59
Log $P_{o/w}$ (MLOGP)	0.22
Log $P_{o/w}$ (SILICOS-IT)	2.55
Consensus Log $P_{o/w}$	2.13

Water Solubility	
Log S (ESOL)	-3.91
Solubility	3.70e-02 mg/ml ; 1.23e-04 mol/l
Class	Soluble
Log S (Ali)	-4.62
Solubility	7.17e-03 mg/ml ; 2.39e-05 mol/l
Class	Moderately soluble
Log S (SILICOS-IT)	-4.52
Solubility	9.07e-03 mg/ml ; 3.02e-05 mol/l
Class	Moderately soluble

Pharmacokinetics	
GI absorption	High
BBB permeant	No
P-gp substrate	No
CYP1A2 inhibitor	Yes
CYP2C19 inhibitor	No
CYP2C9 inhibitor	Yes
CYP2D6 inhibitor	Yes
CYP3A4 inhibitor	Yes
Log K_p (skin permeation)	-6.10 cm/s

Druglikeness	
Lipinski	Yes; 0 violation
Ghose	Yes
Veber	Yes
Egan	Yes
Muegge	Yes
Bioavailability Score	0.55

Medicinal Chemistry	
PAINS	1 alert: catechol_A
Brenk	1 alert: catechol
Leadlikeness	Yes
Synthetic accessibility	3.10

Molecule 9

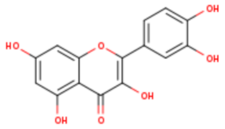

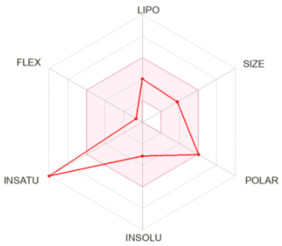

SMILES Oc1cc(O)c2c(c1)oc(c(c2=O)O)c1ccc(c(c1)O)O

Physicochemical Properties	
Formula	C15H10O7
Molecular weight	302.24 g/mol
Num. heavy atoms	22
Num. arom. heavy atoms	16
Fraction Csp3	0.00
Num. rotatable bonds	1
Num. H-bond acceptors	7
Num. H-bond donors	5
Molar Refractivity	78.03
TPSA	131.36 Å²

Lipophilicity	
Log $P_{o/w}$ (iLOGP)	1.63
Log $P_{o/w}$ (XLOGP3)	1.54
Log $P_{o/w}$ (WLOGP)	1.99
Log $P_{o/w}$ (MLOGP)	-0.56
Log $P_{o/w}$ (SILICOS-IT)	1.54
Consensus Log $P_{o/w}$	1.23

Water Solubility	
Log S (ESOL)	-3.16
Solubility	2.11e-01 mg/ml ; 6.98e-04 mol/l
Class	Soluble
Log S (Ali)	-3.91
Solubility	3.74e-02 mg/ml ; 1.24e-04 mol/l
Class	Soluble
Log S (SILICOS-IT)	-3.24
Solubility	1.73e-01 mg/ml ; 5.73e-04 mol/l
Class	Soluble

Pharmacokinetics	
GI absorption	High
BBB permeant	No
P-gp substrate	No
CYP1A2 inhibitor	Yes
CYP2C19 inhibitor	No
CYP2C9 inhibitor	No
CYP2D6 inhibitor	Yes
CYP3A4 inhibitor	Yes
Log K_p (skin permeation)	-7.05 cm/s

Druglikeness	
Lipinski	Yes; 0 violation
Ghose	Yes
Veber	Yes
Egan	Yes
Muegge	Yes
Bioavailability Score	0.55

Medicinal Chemistry	
PAINS	1 alert: catechol_A
Brenk	1 alert: catechol
Leadlikeness	Yes
Synthetic accessibility	3.23

Molecule 10

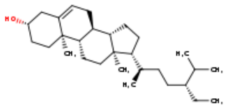

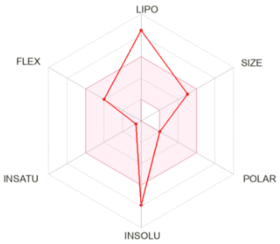

SMILES CC[C@@H](C(C)C)CC[C@H]([C@H]1CC[C@@H]2[C@]1(C)CC[C@H]1[C@H]2CC=C2[C@]1(C)CC[C@@H](C2)O)C

Physicochemical Properties	
Formula	C29H50O
Molecular weight	414.71 g/mol
Num. heavy atoms	30
Num. arom. heavy atoms	0
Fraction Csp3	0.93
Num. rotatable bonds	6
Num. H-bond acceptors	1
Num. H-bond donors	1
Molar Refractivity	133.23
TPSA	20.23 Å²

Lipophilicity	
Log $P_{o/w}$ (iLOGP)	5.05
Log $P_{o/w}$ (XLOGP3)	9.34
Log $P_{o/w}$ (WLOGP)	8.02
Log $P_{o/w}$ (MLOGP)	6.73
Log $P_{o/w}$ (SILICOS-IT)	7.04
Consensus Log $P_{o/w}$	7.24

Water Solubility	
Log S (ESOL)	-7.90
Solubility	5.23e-06 mg/ml ; 1.26e-08 mol/l
Class	Poorly soluble
Log S (Ali)	-9.67
Solubility	8.90e-08 mg/ml ; 2.15e-10 mol/l
Class	Poorly soluble
Log S (SILICOS-IT)	-6.19
Solubility	2.69e-04 mg/ml ; 6.49e-07 mol/l
Class	Poorly soluble

Pharmacokinetics	
GI absorption	Low
BBB permeant	No
P-gp substrate	No
CYP1A2 inhibitor	No
CYP2C19 inhibitor	No
CYP2C9 inhibitor	No
CYP2D6 inhibitor	No
CYP3A4 inhibitor	No
Log K_p (skin permeation)	-2.20 cm/s

Druglikeness	
Lipinski	Yes; 1 violation: MLOGP>4.15
Ghose	No; 3 violations: WLOGP>5.6, MR>130, #atoms>70
Veber	Yes
Egan	No; 1 violation: WLOGP>5.88
Muegge	No; 2 violations: XLOGP3>5, Heteroatoms<2
Bioavailability Score	0.55

Medicinal Chemistry	
PAINS	0 alert
Brenk	1 alert: isolated_alkene
Leadlikeness	No; 2 violations: MW>350, XLOGP3>3.5
Synthetic accessibility	6.30

Molecule 11

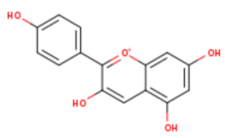

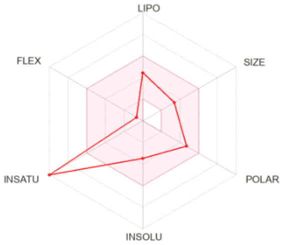

SMILES Oc1ccc(cc1)c1[o+]c2cc(O)cc(c2cc1O)O

Physicochemical Properties	
Formula	C15H11O5+
Molecular weight	271.24 g/mol
Num. heavy atoms	20
Num. arom. heavy atoms	16
Fraction Csp3	0.00
Num. rotatable bonds	1
Num. H-bond acceptors	5
Num. H-bond donors	4
Molar Refractivity	74.15
TPSA	94.06 Å²

Lipophilicity	
Log $P_{o/w}$ (iLOGP)	-2.44
Log $P_{o/w}$ (XLOGP3)	2.29
Log $P_{o/w}$ (WLOGP)	3.20
Log $P_{o/w}$ (MLOGP)	0.86
Log $P_{o/w}$ (SILICOS-IT)	0.73
Consensus Log $P_{o/w}$	0.93

Water Solubility	
Log *S* (ESOL)	-3.49
Solubility	8.77e-02 mg/ml ; 3.23e-04 mol/l
Class	Soluble
Log *S* (Ali)	-3.90
Solubility	3.39e-02 mg/ml ; 1.25e-04 mol/l
Class	Soluble
Log *S* (SILICOS-IT)	-3.24
Solubility	1.58e-01 mg/ml ; 5.81e-04 mol/l
Class	Soluble

Pharmacokinetics	
GI absorption	High
BBB permeant	No
P-gp substrate	Yes
CYP1A2 inhibitor	Yes
CYP2C19 inhibitor	No
CYP2C9 inhibitor	No
CYP2D6 inhibitor	Yes
CYP3A4 inhibitor	No
Log K_p (skin permeation)	-6.33 cm/s

Druglikeness	
Lipinski	Yes; 0 violation
Ghose	Yes
Veber	Yes
Egan	Yes
Muegge	Yes
Bioavailability Score	0.55

Medicinal Chemistry	
PAINS	0 alert
Brenk	1 alert: charged_oxygen_sulfur
Leadlikeness	Yes
Synthetic accessibility	3.04

Molecule 12

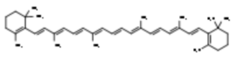

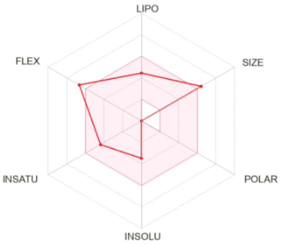

SMILES C/C(=C\C=C\C=C(\C=C\C=C(\C=C\C1=C(C)CCCC1(C)C)/C)/C)/C=C/C=C(/C=C/C1=C(C)CCCC1(C)C)\C

Physicochemical Properties	
Formula	C40H56
Molecular weight	536.87 g/mol
Num. heavy atoms	40
Num. arom. heavy atoms	0
Fraction Csp3	0.45
Num. rotatable bonds	10
Num. H-bond acceptors	0
Num. H-bond donors	0
Molar Refractivity	184.43
TPSA	0.00 Å²

Lipophilicity	
Log $P_{o/w}$ (iLOGP)	-2.44
Log $P_{o/w}$ (XLOGP3)	2.29
Log $P_{o/w}$ (WLOGP)	12.61
Log $P_{o/w}$ (MLOGP)	0.86
Log $P_{o/w}$ (SILICOS-IT)	0.73
Consensus Log $P_{o/w}$	0.93

Water Solubility	
Log *S* (ESOL)	-3.49
Solubility	8.77e-02 mg/ml ; 3.23e-04 mol/l
Class	Soluble
Log *S* (Ali)	-3.90
Solubility	3.39e-02 mg/ml ; 1.25e-04 mol/l
Class	Soluble
Log *S* (SILICOS-IT)	-3.24
Solubility	1.58e-01 mg/ml ; 5.81e-04 mol/l
Class	Soluble

Pharmacokinetics	
GI absorption	High
BBB permeant	No
P-gp substrate	Yes
CYP1A2 inhibitor	Yes
CYP2C19 inhibitor	No
CYP2C9 inhibitor	No
CYP2D6 inhibitor	Yes
CYP3A4 inhibitor	No
Log K_p (skin permeation)	-6.33 cm/s

Druglikeness	
Lipinski	Yes; 0 violation
Ghose	Yes
Veber	Yes
Egan	Yes
Muegge	Yes
Bioavailability Score	0.55

Medicinal Chemistry	
PAINS	0 alert
Brenk	1 alert: charged_oxygen_sulfur
Leadlikeness	Yes
Synthetic accessibility	3.04

Molecule 13

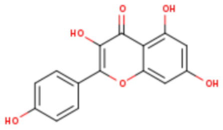

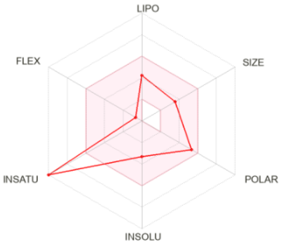

SMILES Oc1ccc(cc1)c1oc2cc(O)cc(c2c(=O)c1O)O

Physicochemical Properties	
Formula	C15H10O6
Molecular weight	286.24 g/mol
Num. heavy atoms	21
Num. arom. heavy atoms	16
Fraction Csp3	0.00
Num. rotatable bonds	1
Num. H-bond acceptors	6
Num. H-bond donors	4
Molar Refractivity	76.01
TPSA	111.13 Å^2

Lipophilicity	
Log $P_{o/w}$ (iLOGP)	1.70
Log $P_{o/w}$ (XLOGP3)	1.90
Log $P_{o/w}$ (WLOGP)	2.28
Log $P_{o/w}$ (MLOGP)	-0.03
Log $P_{o/w}$ (SILICOS-IT)	2.03
Consensus Log $P_{o/w}$	1.58

Water Solubility	
Log *S* (ESOL)	-3.31
Solubility	1.40e-01 mg/ml ; 4.90e-04 mol/l
Class	Soluble
Log *S* (Ali)	-3.86
Solubility	3.98e-02 mg/ml ; 1.39e-04 mol/l
Class	Soluble
Log *S* (SILICOS-IT)	-3.82
Solubility	4.29e-02 mg/ml ; 1.50e-04 mol/l
Class	Soluble

Pharmacokinetics	
GI absorption	High
BBB permeant	No
P-gp substrate	No
CYP1A2 inhibitor	Yes
CYP2C19 inhibitor	No
CYP2C9 inhibitor	No
CYP2D6 inhibitor	Yes
CYP3A4 inhibitor	Yes
Log K_p (skin permeation)	-6.70 cm/s

Druglikeness	
Lipinski	Yes; 0 violation
Ghose	Yes
Veber	Yes
Egan	Yes
Muegge	Yes
Bioavailability Score	0.55

Medicinal Chemistry	
PAINS	0 alert
Brenk	0 alert
Leadlikeness	Yes
Synthetic accessibility	3.14

Molecule 14

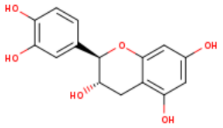

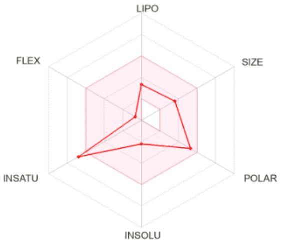

SMILES Oc1cc2O[C@H](c3ccc(c(c3)O)O)[C@H](Cc2c(c1)O)O

Physicochemical Properties	
Formula	C15H14O6
Molecular weight	290.27 g/mol
Num. heavy atoms	21
Num. arom. heavy atoms	12
Fraction Csp3	0.20
Num. rotatable bonds	1
Num. H-bond acceptors	6
Num. H-bond donors	5
Molar Refractivity	74.33
TPSA	110.38 Å^2

Lipophilicity	
Log $P_{o/w}$ (iLOGP)	1.33
Log $P_{o/w}$ (XLOGP3)	0.36
Log $P_{o/w}$ (WLOGP)	1.22
Log $P_{o/w}$ (MLOGP)	0.24
Log $P_{o/w}$ (SILICOS-IT)	0.98
Consensus Log $P_{o/w}$	0.83

Water Solubility	
Log *S* (ESOL)	-2.22
Solubility	1.74e+00 mg/ml ; 5.98e-03 mol/l
Class	Soluble
Log *S* (Ali)	-2.24
Solubility	1.66e+00 mg/ml ; 5.72e-03 mol/l
Class	Soluble
Log *S* (SILICOS-IT)	-2.14
Solubility	2.09e+00 mg/ml ; 7.19e-03 mol/l
Class	Soluble

Pharmacokinetics	
GI absorption	High
BBB permeant	No
P-gp substrate	Yes
CYP1A2 inhibitor	No
CYP2C19 inhibitor	No
CYP2C9 inhibitor	No
CYP2D6 inhibitor	No
CYP3A4 inhibitor	No
Log K_p (skin permeation)	-7.82 cm/s

Druglikeness	
Lipinski	Yes; 0 violation
Ghose	Yes
Veber	Yes
Egan	Yes
Muegge	Yes
Bioavailability Score	0.55

Medicinal Chemistry	
PAINS	1 alert: catechol_A
Brenk	1 alert: catechol
Leadlikeness	Yes
Synthetic accessibility	3.50

Molecule 15

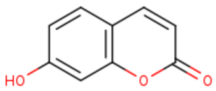

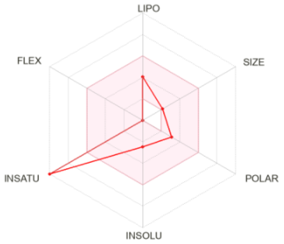

SMILES Oc1ccc2c(c1)oc(=O)cc2

Physicochemical Properties	
Formula	C9H6O3
Molecular weight	162.14 g/mol
Num. heavy atoms	12
Num. arom. heavy atoms	10
Fraction Csp3	0.00
Num. rotatable bonds	0
Num. H-bond acceptors	3
Num. H-bond donors	1
Molar Refractivity	44.51
TPSA	50.44 Å²

Lipophilicity	
Log $P_{o/w}$ (iLOGP)	1.44
Log $P_{o/w}$ (XLOGP3)	1.58
Log $P_{o/w}$ (WLOGP)	1.50
Log $P_{o/w}$ (MLOGP)	1.04
Log $P_{o/w}$ (SILICOS-IT)	1.97
Consensus Log $P_{o/w}$	1.51

Water Solubility	
Log *S* (ESOL)	-2.46
Solubility	5.66e-01 mg/ml ; 3.49e-03 mol/l
Class	Soluble
Log *S* (Ali)	-2.25
Solubility	9.12e-01 mg/ml ; 5.62e-03 mol/l
Class	Soluble
Log *S* (SILICOS-IT)	-3.03
Solubility	1.53e-01 mg/ml ; 9.42e-04 mol/l
Class	Soluble

Pharmacokinetics	
GI absorption	High
BBB permeant	Yes
P-gp substrate	No
CYP1A2 inhibitor	Yes
CYP2C19 inhibitor	No
CYP2C9 inhibitor	No
CYP2D6 inhibitor	No
CYP3A4 inhibitor	No
Log K_p (skin permeation)	-6.17 cm/s

Druglikeness	
Lipinski	Yes; 0 violation
Ghose	No; 1 violation: #atoms<20
Veber	Yes
Egan	Yes
Muegge	No; 1 violation: MW<200
Bioavailability Score	0.55

Medicinal Chemistry	
PAINS	0 alert
Brenk	1 alert: cumarine
Leadlikeness	No; 1 violation: MW<250
Synthetic accessibility	2.56

Molecule 16

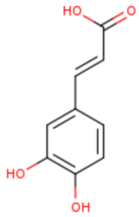

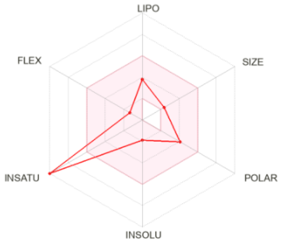

SMILES OC(=O)/C=C/c1ccc(c(c1)O)O

Physicochemical Properties	
Formula	C9H8O4
Molecular weight	180.16 g/mol
Num. heavy atoms	13
Num. arom. heavy atoms	6
Fraction Csp3	0.00
Num. rotatable bonds	2
Num. H-bond acceptors	4
Num. H-bond donors	3
Molar Refractivity	47.16
TPSA	77.76 Å²

Lipophilicity	
Log $P_{o/w}$ (iLOGP)	0.97
Log $P_{o/w}$ (XLOGP3)	1.15
Log $P_{o/w}$ (WLOGP)	1.09
Log $P_{o/w}$ (MLOGP)	0.70
Log $P_{o/w}$ (SILICOS-IT)	0.75
Consensus Log $P_{o/w}$	0.93

Water Solubility	
Log *S* (ESOL)	-1.89
Solubility	2.32e+00 mg/ml ; 1.29e-02 mol/l
Class	Very soluble
Log *S* (Ali)	-2.38
Solubility	7.55e-01 mg/ml ; 4.19e-03 mol/l
Class	Soluble
Log *S* (SILICOS-IT)	-0.71
Solubility	3.51e+01 mg/ml ; 1.95e-01 mol/l
Class	Soluble

Pharmacokinetics	
GI absorption	High
BBB permeant	No
P-gp substrate	No
CYP1A2 inhibitor	No
CYP2C19 inhibitor	No
CYP2C9 inhibitor	No
CYP2D6 inhibitor	No
CYP3A4 inhibitor	No
Log K_p (skin permeation)	-6.58 cm/s

Druglikeness	
Lipinski	Yes; 0 violation
Ghose	Yes
Veber	Yes
Egan	Yes
Muegge	No; 1 violation: MW<200
Bioavailability Score	0.56

Medicinal Chemistry	
PAINS	1 alert: catechol_A
Brenk	2 alerts: catechol, michael_acceptor_1
Leadlikeness	No; 1 violation: MW<250
Synthetic accessibility	1.81

Molecule 17

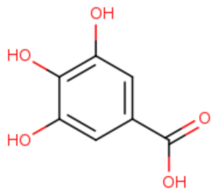

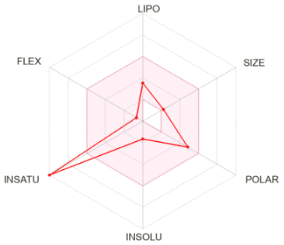

SMILES OC(=O)c1cc(O)c(c(c1)O)O

Physicochemical Properties	
Formula	C7H6O5
Molecular weight	170.12 g/mol
Num. heavy atoms	12
Num. arom. heavy atoms	6
Fraction Csp3	0.00
Num. rotatable bonds	1
Num. H-bond acceptors	5
Num. H-bond donors	4
Molar Refractivity	39.47
TPSA	97.99 Å²

Lipophilicity	
Log $P_{o/w}$ (iLOGP)	0.21
Log $P_{o/w}$ (XLOGP3)	0.70
Log $P_{o/w}$ (WLOGP)	0.50
Log $P_{o/w}$ (MLOGP)	-0.16
Log $P_{o/w}$ (SILICOS-IT)	-0.20
Consensus Log $P_{o/w}$	0.21

Water Solubility	
Log *S* (ESOL)	-1.64
Solubility	3.90e+00 mg/ml ; 2.29e-02 mol/l
Class	Very soluble
Log *S* (Ali)	-2.34
Solubility	7.86e-01 mg/ml ; 4.62e-03 mol/l
Class	Soluble
Log *S* (SILICOS-IT)	-0.04
Solubility	1.55e+02 mg/ml ; 9.10e-01 mol/l
Class	Soluble

Pharmacokinetics	
GI absorption	High
BBB permeant	No
P-gp substrate	No
CYP1A2 inhibitor	No
CYP2C19 inhibitor	No
CYP2C9 inhibitor	No
CYP2D6 inhibitor	No
CYP3A4 inhibitor	Yes
Log K_p (skin permeation)	-6.84 cm/s

Druglikeness	
Lipinski	Yes; 0 violation
Ghose	No; 2 violations: MR<40, #atoms<20
Veber	Yes
Egan	Yes
Muegge	No; 1 violation: MW<200
Bioavailability Score	0.56

Medicinal Chemistry	
PAINS	1 alert: catechol_A
Brenk	1 alert: catechol
Leadlikeness	No; 1 violation: MW<250
Synthetic accessibility	1.22

Molecule 18

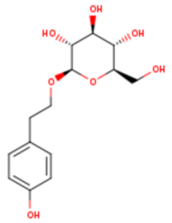

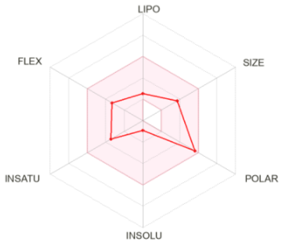

SMILES OC[C@H]1O[C@@H](OCCc2ccc(cc2)O)[C@@H]([C@H]([C@@H]1O)O)O

Physicochemical Properties	
Formula	C14H20O7
Molecular weight	300.30 g/mol
Num. heavy atoms	21
Num. arom. heavy atoms	6
Fraction Csp3	0.57
Num. rotatable bonds	5
Num. H-bond acceptors	7
Num. H-bond donors	5
Molar Refractivity	71.78
TPSA	119.61 Å²

Lipophilicity	
Log $P_{o/w}$ (iLOGP)	1.04
Log $P_{o/w}$ (XLOGP3)	-1.05
Log $P_{o/w}$ (WLOGP)	-1.25
Log $P_{o/w}$ (MLOGP)	-1.22
Log $P_{o/w}$ (SILICOS-IT)	-0.48
Consensus Log $P_{o/w}$	-0.59

Water Solubility	
Log *S* (ESOL)	-0.92
Solubility	3.60e+01 mg/ml ; 1.20e-01 mol/l
Class	Very soluble
Log *S* (Ali)	-0.97
Solubility	3.19e+01 mg/ml ; 1.06e-01 mol/l
Class	Very soluble
Log *S* (SILICOS-IT)	-0.44
Solubility	1.08e+02 mg/ml ; 3.61e-01 mol/l
Class	Soluble

Pharmacokinetics	
GI absorption	High
BBB permeant	No
P-gp substrate	No
CYP1A2 inhibitor	No
CYP2C19 inhibitor	No
CYP2C9 inhibitor	No
CYP2D6 inhibitor	No
CYP3A4 inhibitor	No
Log K_p (skin permeation)	-8.88 cm/s

Druglikeness	
Lipinski	Yes; 0 violation
Ghose	No; 1 violation: WLOGP<-0.4
Veber	Yes
Egan	Yes
Muegge	Yes
Bioavailability Score	0.55

Medicinal Chemistry	
PAINS	0 alert
Brenk	0 alert
Leadlikeness	Yes
Synthetic accessibility	4.26
