# Supplementary material for: Xinnaoxin tablets ameliorate high-altitude polycythemia-associated cardiac injury by regulating the NF-κB, MAPK, and PI3K/AKT signaling pathways
Source: Front Pharmacol. 2026 May 28;17:1754806. doi: 10.3389/fphar.2026.1754806 (PMC13253415; doi:10.3389/fphar.2026.1754806)
Supplement: Supplementary file 9 [file DataSheet8.pdf]

No.	Disease-related targets	No.	Disease-related targets
1	HMGN2	51	SLC30A10
2	HMGB4	52	APOA1
3	PKLR	53	HBA1
4	FCER1G	54	MPL
5	EGLN1	55	HBA2
6	GLS	56	LOC107133510
7	HDLBP	57	ACE
8	CAV3	58	TET2
9	KNG1	59	JAK1
10	P3H2	60	LOC106099062
11	SOD3	61	KIT
12	HMGB2	62	LOC110006319
13	SLC1A3	63	HIF1A
14	HMGA1	64	IL6
15	HMGN3	65	CD177
16	PDE7B	66	INS
17	CFTR	67	STAT3
18	LPL	68	CRP
19	TOX	69	BRCA1
20	GPIHBP1	70	MIR150
21	SLC1A1	71	NOS3
22	JAK2	72	REN
23	SLC1A2	73	APOE
24	MS4A2	74	F2
25	SCNN1A	75	CALR
26	HMGA2	76	TNF
27	SCARB1	77	IGF1
28	HMGB1	78	AGTR1
29	TOX4	79	ALB
30	HMGN2P46	80	AGT
31	LIPC	81	EDN1
32	HMG20A	82	VEGFA
33	SCNN1G	83	HFE
34	SCNN1B	84	ABL1
35	TOX3	85	BCL2
36	CETP	86	AKT1
37	HMG20B	87	F5
38	SLC1A6	88	MYC
39	TOX2	89	MTHFR
40	HMGN1	90	GATA1
41	GGT2P	91	FGFR3
42	HMGB3	92	STAT5A
43	VHL	93	THPO
44	LOC107303340	94	CSF3
45	EPOR	95	KITLG
46	EPAS1	96	NPPA
47	HBB	97	ASXL1
48	EPO	98	TGFB1
49	INSL6	99	APOC3
50	TP53	100	LEP

No.	Disease-related targets
101	IFNA1
102	NTRK1
103	TFRC
104	SELP
105	F3
106	IL1B
107	LOC106804612
108	IL3
109	APOB
110	CTNNB1
111	ESR1
112	THBD
113	CSF2
114	G6PD
115	ITGB3
116	SETD2
117	HAMP
118	IFNG
119	SDHB
120	MIR155
121	CXCL8
122	TF
123	CBS
124	VWF
125	SDHD
126	SOD1
127	GNAS
128	STAT5B
129	IL10
130	SLC2A1
131	NR3C1
132	AR
133	HLA-DRB1
134	SERPINE1
135	EGLN3
136	CERNA3
137	MIR451A
138	TTR
139	SLC4A1
140	PIK3CD
141	PTPN6
142	ACTB
143	GP1BA
144	FLT1
145	PTPRC
146	EP300
147	PPARA
148	MIR125A
149	EGLN2
150	MMP9

No.	Disease-related targets
151	ADRB2
152	PF4
153	SELE
154	GATA2
155	ABCB1
156	TIMP1
157	CYP3A4
158	IL4
159	ACP1
160	ERCC2
161	CXCR4
162	MPO
163	POMC
164	FN1
165	CCL2
166	IL2
167	CP
168	EDNRB
169	CD36
170	MT-ND1
171	TH
172	TACR1
173	PRL
174	DMD
175	GAPDH
176	SERPINC1
177	ACVRL1
178	SOD2-OT1
179	ICAM1
180	SOX2
181	PIEZO1
182	NFKB1
183	IL2RA
184	IL13
185	MT-CO1
186	MIR210
187	ELOC
188	TG
189	CYP17A1
190	SDHC
191	PTH
192	UGT1A1
193	GH1
194	HP
195	NPY
196	SERPINA1
197	AKT3
198	TLR4
199	IL1RN
200	GATA3

No.	Disease-related targets
201	SLC11A2
202	FGF2
203	CD55
204	LOX
205	BMPR2
206	CAT
207	SST
208	VCAM1
209	DNMT1
210	GSR
211	PPBP
212	MME
213	BMP2
214	CD34
215	NLRP3
216	GYPA
217	COL4A3
218	MIR146B
219	CMTS
220	VEGFC
221	MIR144
222	MB
223	FGA
224	MIR142
225	HLA-A
226	BRD4
227	CD40LG
228	ACE2
229	MIR146A
230	NFE2
231	APOA4
232	HSPA4
233	ACHE
234	SLC16A1
235	AURKA
236	GPT
237	LDHA
238	CD4
239	GNRH1
240	GPT2
241	MDH2
242	PLAT
243	BCAM
244	CASP8
245	IL1R1
246	S100B
247	CYBA
248	PHOX2B
249	UCP2
250	IL1A
251	PTGIS
252	PGF
253	ADAMTS13
254	GSTM1
255	PLG
256	IL18
257	ALAS2
258	MIF
259	ITGAM
260	COL6A1
261	BMP4
262	POU5F1
263	ABCG2
264	FGB
265	MIRLET7C
266	ARG1
267	HSP90AA1
268	ESR2
269	CYP2E1
270	ARNT
271	CXCL12
272	HIF1AN
273	SELPLG
274	H6PD
275	TPO
276	GCG
277	PKM
278	SENP1
279	SELL
280	IL2RG
281	HBG2
282	ITGA2B
283	MYB
284	RAP1A
285	SLC40A1
286	ANGPT2
287	CALCA
288	GSTT1
289	PECAM1
290	FTO
291	HLA-G
292	GAST
293	KEL
294	CD8A
295	SCARB2
296	KCNN4
297	UGT1A6
298	KLF1
299	ANXA5
300	SLC29A1

No.	Disease-related targets
301	BCL11A
302	MRTFA
303	FGF10
304	ANK1
305	UGT1A4
306	KMT2C
307	CD19
308	F7
309	CS
310	CD163
311	UGT1A10
312	UGT1A7
313	UGT1A8
314	FAS-AS1
315	ANGPT1
316	HDAC9
317	MIR486-1
318	HSP90AB1
319	ENO2
320	MAPK8
321	EPB41
322	PWAR1
323	MIR181B1
324	PIK3C2A
325	TRH
326	CARD14
327	KLF4
328	PSMA7
329	TMPRSS6
330	CBLIF
331	LDHB
332	FER
333	CSNK2B
334	CKM
335	CD47
336	NANOG
337	CDA
338	PPP1R2P1
339	LIN28A
340	FLT3LG
341	HBS1L
342	PROCR
343	PWAR4
344	ZFPM1
345	TAL1
346	PAPPA2
347	S100A12
348	YTHDF1
349	ATP5IF1
350	MPP1

No.	Disease-related targets
351	WDR77
352	AHSP
353	HOXB4
354	LMO2
355	NACA
356	ICAM4
357	LINC02228
358	NOX1
359	MT-CO1
360	HBB
361	HBA1
