# Supplementary material for: Xinnaoxin tablets ameliorate high-altitude polycythemia-associated cardiac injury by regulating the NF-κB, MAPK, and PI3K/AKT signaling pathways
Source: Front Pharmacol. 2026 May 28;17:1754806. doi: 10.3389/fphar.2026.1754806 (PMC13253415; doi:10.3389/fphar.2026.1754806)
Supplement: Supplementary file 10 [file DataSheet10.pdf]

name	Betweenn	Closeness	Degree	Eigenvecto	LAC	Network
ALB	93.35472	0.966667	28	0.270662	13.71429	27.89181
TNF	93.35472	0.966667	28	0.270662	13.71429	27.89181
AKT1	26.93885	0.828571	23	0.250958	14.43478	21.51682
ACTB	19.65234	0.805556	22	0.246721	14.63636	20.58038
TGFB1	16.74941	0.783784	21	0.237488	14.28571	19.20535
BCL2	8.997027	0.763158	20	0.236264	14.9	18.50022
CTNNB1	34.54589	0.763158	20	0.230293	14.2	18.38771
ESR1	18.35142	0.763158	20	0.229879	14.1	18.15944
ICAM1	22.19836	0.763158	20	0.21603	12.4	16.8514
HSP90AA1	33.01639	0.74359	19	0.214105	12.84211	16.70894
MYC	4.572966	0.725	18	0.218608	14.33333	16.74797
VCAM1	17.29689	0.707317	17	0.186046	10.94118	13.91964
NOS3	16.57315	0.707317	17	0.191594	11.29412	13.82652
NR3C1	7.790976	0.690476	16	0.188648	11.625	13.51667
ESR2	1.207692	0.674419	15	0.189944	13.06667	14.13095
CASP8	1.680109	0.674419	15	0.189289	12.8	14.07143
MAPK8	1.26337	0.674419	15	0.192645	12.93333	13.85714
AR	3.357143	0.674419	15	0.184847	12.4	13.75
DNMT1	0.309524	0.644444	13	0.169197	11.69231	12.66667
SELE	3.57381	0.617021	12	0.136469	9	10.37305
F3	5.904762	0.604167	11	0.110583	7.454545	9.575
JAK1	0.133333	0.604167	10	0.132755	8.8	9.777778
CYP3A4	4.752381	0.58	9	0.100891	5.777778	7.125
F2	5.304762	0.58	9	0.079384	5.333333	6.875
MIF	0.667647	0.58	8	0.10147	6.25	7.142857
ADRB2	0.166667	0.557692	7	0.093118	5.714286	6.666667
F7	0	0.537037	5	0.052156	4	5
MB	0.285714	0.537037	5	0.052479	3.6	4.5
GSTM1	0	0.527273	4	0.049663	3	4
CSNK2B	0	0.453125	2	0.025339	1	2

	Betweenn	Closeness	Degree	Eigenvect	LAC	Network
TOP20	ALB	ALB	ALB	ALB	BCL2	ALB
	TNF	TNF	TNF	TNF	ACTB	TNF
	CTNNB1	AKT1	AKT1	AKT1	AKT1	AKT1
	HSP90AA	ACTB	ACTB	ACTB	MYC	ACTB
	AKT1	TGFB1	TGFB1	TGFB1	TGFB1	TGFB1
	ICAM1	CTNNB1	CTNNB1	BCL2	CTNNB1	BCL2
	ACTB	ICAM1	ICAM1	CTNNB1	ESR1	CTNNB1
	ESR1	ESR1	ESR1	ESR1	ALB	ESR1
	VCAM1	BCL2	BCL2	MYC	TNF	ICAM1
	TGFB1	HSP90AA	HSP90AA	ICAM1	ESR2	MYC
	NOS3	MYC	MYC	HSP90AA	MAPK8	HSP90AA1
	BCL2	VCAM1	VCAM1	MAPK8	HSP90AA	ESR2
	NR3C1	NOS3	NOS3	NOS3	CASP8	CASP8
	F3	NR3C1	NR3C1	ESR2	ICAM1	VCAM1
	F2	AR	AR	CASP8	AR	MAPK8
	CYP3A4	CASP8	CASP8	NR3C1	DNMT1	NOS3
	MYC	MAPK8	MAPK8	VCAM1	NR3C1	AR
	SELE	ESR2	ESR2	AR	NOS3	NR3C1
	AR	DNMT1	DNMT1	DNMT1	VCAM1	DNMT1
